# The impact of diarrhoea measurement methods for under 5s in low‐ and middle‐income countries on estimated diarrhoea rates at the population level: A systematic review and meta‐analysis of methodological and primary empirical studies

**DOI:** 10.1111/tmi.13739

**Published:** 2022-03-07

**Authors:** Ryan Rego, Samuel Watson, Paramjit Gill, Richard Lilford

**Affiliations:** ^1^ Center for Global Health Equity University of Michigan at Ann Arbor Ann Arbor Michigan USA; ^2^ Institute for Applied Health Research University of Birmingham Birmingham UK; ^3^ Center for Global Health Warwick Medical School University of Warwick Coventry UK

**Keywords:** child health, diarrhoea, epidemiology, surveillance, WASH

## Abstract

**Objective:**

We systematically reviewed all studies published between 2000 and June 2021 that estimated under 5 diarrhoea rates in low‐ and middle‐income countries and extracted data on diarrhoea rates, measurement methods and reactivity.

**Methods:**

We summarised data from studies that performed direct comparisons of methods, and indirectly compared studies which utilised only one method using meta‐regression to determine the association between methods and estimated diarrhoea rates.

**Results:**

In total, 288 studies met our inclusion criteria: 4 direct comparisons and 284 studies utilising only one measurement method. Meta‐regression across all studies showed that diarrhoea rates were sensitive to method of measurement. We estimated that passive surveillance methods were associated with a 97% lower estimated rate than active surveillance (IRR = 0.03, 95% CI [0.02, 0.06]). Among active surveillance studies, a doubling of recall period was associated with a 48% lower rate (IRR = 0.52 [0.46, 0.60]), while decreased questioning frequency was associated with a higher estimated rate: at the extreme, one time questioning yielded an over 4× higher rate than daily questioning (IRR = 4.22 [2.73, 6.52]).

**Conclusions:**

Estimated diarrhoea rates are sensitive to their measurement methods. There is a need for a standardisation of diarrhoea measurement methods, and for the use of other outcomes in the measurement of population‐level gastrointestinal health.

## INTRODUCTION

Effective surveillance of diarrhoea in children under 5 at the population level is required to track outbreaks, allocate public health resources and evaluate water, sanitation and hygiene (WASH) interventions [1]. However, the choice of method used to ascertain whether an episode of diarrhoea has occurred has been hypothesised to impact estimated diarrhoea rates [2, 3]. If true, the results of observational and intervention studies may differ by method of measurement, thereby obscuring the effects of the explanatory variable(s) of interest. Since diarrhoea is one of the biggest causes of death in children, this is a methodological point of considerable practical importance for surveillance, evaluation of interventions and establishing the true burden of diarrhoea‐associated morbidity and mortality. The two most common methods of diarrhoea surveillance at the population level, passive and active surveillance, take different approaches. Passive surveillance relies on data collected from health facilities and therefore excludes all children with diarrhoea who do not attend a facility. Passive surveillance estimates are therefore skewed towards more severe disease and away from marginalised groups such as slum dwellers, refugees and migrants, who are less likely to visit health facilities and who are more likely to visit informal facilities than non‐marginalised groups [4, 5, 6, 7, 8, 9]. Passive surveillance is a useful, inexpensive tool to detect new outbreaks of severe diseases such as cholera, but is arguably less useful as an epidemiological tool for the measurement of population‐level diarrhoea rates or in trials of WASH interventions.

Active surveillance, based on door‐to‐door surveys, provides a more complete report of diarrhoea rates than passive surveillance, but may also be subject to measurement error and bias [4]. Carers may forget events that happened in the past, particularly during longer lengths of recall [10, 11]. They may also have a poor understanding of what diarrhoea is [12]. UNICEF and the Demographics and Health Surveys (DHS) programme have recommended a method based on asking carers if their child has had three or more loose or watery stools in any 24‐h period within the previous 14 days [13]. However, this method is by no means universally applied. In addition to concerns over measurement error, concern has been expressed that diarrhoea rates may be subjective to bias due to ‘reactivity’, where despite any true clinical difference, people report different diarrhoea rates for psychological reasons. For example, they may be the beneficiaries of an intervention and not want to appear ungrateful; not want to answer in a way that is not socially desirable or be guided subliminally or non‐verbally by the surveyors (e.g. if the question is asked in a way which steers the respondents towards a certain answer) [14, 15]. As such, reactivity creates a particular concern for evaluations of WASH interventions.

In order to examine the association between the method used in diarrhoea measurement and diarrhoea rates, we conducted a systematic review of all studies published between 2000 and June 2021 that report under five diarrhoea rates in low‐ and middle‐income countries (LMICs). We examined studies that perform direct ‘head‐to‐head’ comparisons of different methods in order to estimate differences in diarrhoea rates by method. However, we found only four such studies. We therefore obtained studies that used only one measurement method so that we could compare the estimated diarrhoea rates of each method across studies indirectly by means of meta‐analytical methods [16, 17].

The aims of the systematic review and meta‐analysis were to determine: (1) the frequency of the use of the different diarrhoea measurement methods; (2) the association between passive and active surveillance methods and estimated diarrhoea rates; (3) the association between recall periods, questioning frequencies and prospective (diary) versus retrospective recall on estimated diarrhoea rates among active surveillance studies; and (4) the extent of reactivity in diarrhoea measurement.

## METHODS

### Search strategy and selection criteria

We conducted a systematic review of studies published between 2000 and June 2021 that made quantitative measurements of diarrhoea rates among children under the age of 5 in LMICs (as defined by OECD) [18]. The search strategy aimed to capture any study that estimated diarrhoea rates among under 5s, including both studies that performed direct ‘head‐to‐head’ comparisons of methods, and studies estimating diarrhoea rates using only one method that we can compare indirectly. As we are only interested in population‐level diarrhoea rates, we excluded studies that were not designed to capture population‐level diarrhoea rates, such as studies which measured hospital acquired infection, clinical trials in which diarrhoea was an adverse drug event and case–control studies in which diarrhoea was the case. Importantly, these exclusions do not include WASH trials which took place in the community setting. Studies were restricted to English or French (Table 1).

**TABLE 1:** Inclusion and exclusion criteria

Inclusion criteria	Exclusion criteria
Reports diarrhoea rates among under 5s (i.e. incidence or prevalence)	Measures hospital‐acquired infection
Takes place in LMICs	Clinical trials in which diarrhoea was an adverse drug event
Published between 2000 and June 2021	Case–control studies in which diarrhoea is the case
English or French language	

We searched the MEDLINE, Embase and PubMed databases for studies matching the inclusion and exclusion criteria. The search string (Appendix 1) included keywords relating to diarrhoea and population‐level disease measurement. The string then restricted the results to human studies and studies in LMICs.

SW and RR independently screened each title and abstract, and any disagreements were resolved through discussion with RL. Full texts were screened in a similar manner. Where full texts were not available, we requested the article from the University of Warwick's Article Reach Service. We excluded unavailable studies and duplicate studies. In the event that multiple studies used the same data source, we selected a random study for inclusion.

### Data extraction

RR extracted the data, with SW duplicating data extraction for random 15% of the included studies. The random 15% extracted by SW matched completely with the extraction by RR. As a single reported study may have included several independent reports of diarrhoea rates, we treated each report as separate ‘observations’ within one study. This would not only apply to all the direct studies but also arose in indirect studies when conducted in more than one site, included multiple rounds of data collection and/or was a trial with multiple arms. For example, a two‐armed trial which estimated diarrhoea rates at baseline and end‐line yielded four observations.

Data extracted (Table 2) included participant demographics; study design, if the study was an observational study using primary data (including non‐randomised trials), an observational study using secondary data or a randomised control trial; diarrhoea rates; measurement methods and if the study was a direct comparison of methods. We defined direct comparison studies as those which included at least two separate arms, each with a different method of diarrhoea measurement (including altering recall period or questioning frequency) that compare estimated diarrhoea rates between each arm.

**TABLE 2:** Data extracted for each observation

Variable	Units (if applicable)	Categorisation (if categorised)
Authors	NA	NA
Study title	NA	NA
Year of publication	Years	NA
Year of data collection	Years	NA
Mean participant age	Years (with standard error)	NA
Participant sex breakdown	% Female	NA
Geography	Urban, rural or mixed	NA
Country	NA	Regions as defined by the United Nations Development Programme (UNDP) [19]
Intervention (for randomised control trials [RCTs])	NA	WASH, Health or Nutrition
Effectiveness of intervention (for RCTs)	Incidence risk ratio	NA
Diarrhoea rate (in incidence)	Episodes per child year (converted if necessary)	NA
Measurement type	NA	Passive, retrospective active, prospective (diary) active
Recall period	Days	NA
Questioning frequency	Days	Daily, weekly, monthly or annually or longer
Questioning type	NA	Pictorial, verbal or other
Sample size (observed cases for passive surveillance studies)	NA	NA
Study design	NA	Observational (primary), observational (secondary), RCT (broken down by arm)
Direct comparison of methods	NA	Yes/no

### Data analysis

We indirectly compared the diarrhoea rates from included observations, including those from studies that performed direct comparisons of measurement methods, and studies using only a single method of measurement. We summarised the key variables in each individual observation, including estimated diarrhoea rates, measurement type, year and region (as defined by UNDP). We estimated a hierarchical meta‐regression model for log diarrhoea rate (incidence in episodes per child year), adjusting for region and time (in years) and interactions between time and region. We also adjusted for study design, including a categorical variable with levels: (1) observational studies which use primary data sources; (2) observational studies which use secondary data sources; (3) RCT intervention arms before the intervention, or the control arm (if reported) and (4) RCT experimental arm after the intervention. The model was estimated in StataSE Version 15 using generalised least squares with random effects at the study level, to account for within‐study correlation, and weighting by the study sample size [19, 20].

We estimated two separate meta‐regression models. The first iteration included the observations from both passive and active surveillance studies to estimate the effect of surveillance types (passive/active) on estimated diarrhoea rates, and as such included a dummy variable for surveillance type. We also estimated pooled temporal and regional trends from this model.

The second iteration included only observations from active surveillance studies to examine the effects of variables exclusive to active surveillance studies on estimated diarrhoea rates. These included variables for recall period (as a continuous numeric term in days), and questioning frequency and recall type (both as categorical variables). Furthermore, any other variations found between active surveillance studies, including reactivity and questioning type (e.g. verbal or pictorial), were included (Table 3).

**TABLE 3:** Summary table of the 671 observations included in the systematic review, with average estimated diarrhoea rates for key variables

		Passive *n* (% of total)	Prospective (diary) active *n* (% of total)	Retrospective active *n* (% of total)	Total	Average estimated diarrhoea rate (episodes per child year)
	Total	25	13	633	671	6.75
Data collection year	2016–2018	1 (2.9%)	0 (0%)	33 (97.1%)	34	7.08
	2011–2015	12 (6.8%)	2 (1.1%)	163 (92.1%)	177	6.63
	2006–2010	6 (3.2%)	2 (1.1%)	180 (95.7%)	188	6.60
	2001–2005	4 (3.4%)	2 (1.7%)	112 (94.9%)	118	7.80
	1996–2000	2 (2.5%)	1 (1.3%)	77 (96.2%)	80	6.15
	1991–1995	0 (0%)	0 (0%)	38 (100%)	38	5.99
	1986–1990	0 (0%)	3 (42.9%)	4 (57.2%)	7	3.54
	Missing data	0 (0%)	3 (10.3%)	26 (89.7%)	29	7.46
Region	Sub‐Saharan Africa	5 (1.9%)	3 (1.1%)	257 (97.0%)	265	6.32
	North Africa and the Middle East	0 (0%)	0 (0%)	23 (93.4%)	23	6.56
	Central and Southern Asia	3 (1.7%)	2 (1.1%)	175 (97.2%)	180	9.41
	Eastern and South‐East Asia	13 (21.0%)	4 (6.4%)	45 (72.6%)	62	3.20
	Oceania	0 (0%)	0 (0%)	1 (100.0%)	1	4.02
	The Americas	5 (3.6%)	3 (2.1%)	132 (94.3%)	140	5.74
Study design	Observational (primary)	19 (6.5%)	11 (3.7%)	264 (89.8%)	294	8.21
	Observational (secondary)	6 (3.2%)	0 (0%)	183 (96.8%)	189	3.87
	RCT (baseline or control)	0 (0%)	1 (1.1%)	93 (98.9%)	94	8.37
	RCT (experimental post‐intervention)	0 (0%)	1 (1.1%)	93 (98.9%)	94	6.32
Geography	Mixed	14 (5.8%)	2 (0.8%)	226 (93.4%)	242	4.02
	Rural	4 (1.8%)	4 (1.8%)	212 (96.3%)	220	10.55
	Urban	4 (3.1%)	7 (5.5%)	116 (91.3%)	127	5.15
	Missing data	3 (3.7%)	0 (0%)	79 (96.4%)	82	6.99
Questioning frequency	Daily	NA	0 (0%)	55 (100%)	55	4.68
	Weekly	NA	9 (9.5%)	86 (90.5%)	95	4.49
	Monthly	NA	1 (1.3%)	77 (98.8%)	78	13.47
	Annual+	NA	0 (0%)	30 (100%)	30	5.43
	One off	NA	3 (0.8%)	385 (99.2%)	388	6.76
Recall period	1–3 days	NA	1 (1.6%)	60 (98.4%)	61	25.88
	4–14 days	NA	11 (2.1%)	520 (97.9%)	531	5.22
	15–30 days	NA	1 (2.6%)	37 (97.4%)	38	3.94
	31–90 days	NA	0 (0%)	13 (100%)	13	1.00
	91+ days	NA	0 (0%)	3 (12.5%)	3	1.83

## RESULTS

### Study identification

**FIGURE 1:**
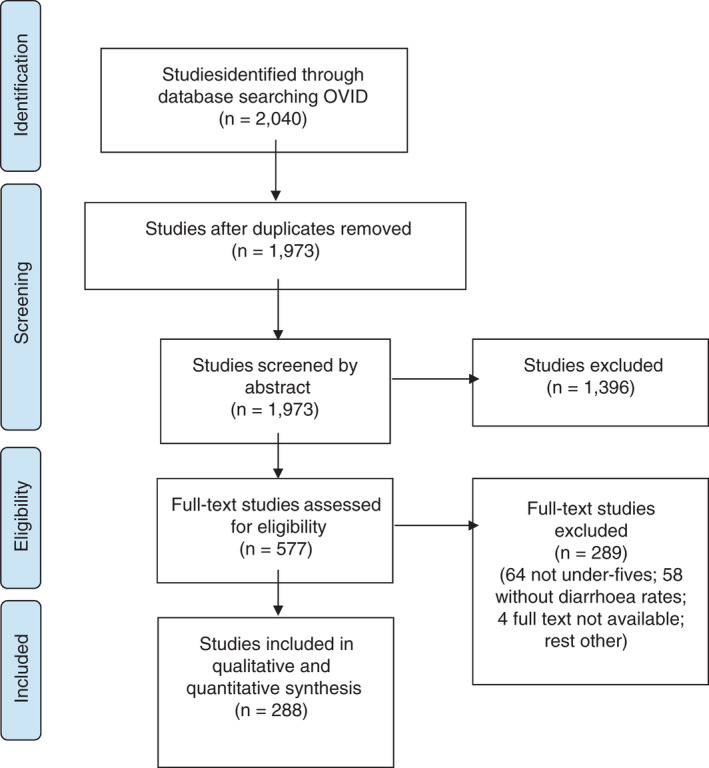
PRISMA diagram of the systematic review

We identified 2040 studies in total, which was reduced to 1973 after duplicates were removed. Abstract and title screening yielded 577 studies, with a further 289 excluded after full‐text review. Common reasons for exclusion included not presenting data on under 5s (*n* = 64), not including data on diarrhoea rates (*n* = 58), and not being able to obtain the full text (*n* = 4). Thus, 288 full‐text studies (Appendix 2) were included in the final review (Figure 1).

### Study characteristics

We first describe the characteristics of the described studies, such as observations per study, data collection method, geography and others. As stated, many studies included more than one observation of diarrhoea rate, arising from observations at different time points, being a trial with two or more arms, or data collection in more than one location. Appendix 3 presents information on the number of observations per study. We identified 671 separate observations of population‐level diarrhoea rates, and these constitute our denominator. In total, there were 646 (96%) active surveillance observations and 25 (4%) passive surveillance observations. Of the 646 active surveillance observations, 633 (94%) were retrospective, while 13 (2%) were prospective (a diary kept by the carer). Of the observations, 354 (56%) used a 14‐day recall period, as recommended by UNICEF and the Demographic and Health Survey (DHS) programme. Of the 188 observations which came from randomised control trials (RCTs), 53 (28%) used a 14‐day recall period and 95 (51%) used a 7‐day recall period. Furthermore, of the observations from RCTs, 21 (12%) questioned daily, 56 (30%) questioned weekly and 49 (26%) questioned biweekly.

By region (as defined by the UNDP), sub‐Saharan Africa was the setting for the largest number of observations (265; 40%), followed by The Americas (140; 21%), Central and Southern Asia (180; 27%), East and South‐East Asia (62; 9%), North Africa and the Middle East (23; 3%) and finally Oceania (1; 0%). Rural (212; 32%) and mixed geography (226; 34%) areas provided more observations than urban areas (116; 17%).

None of the included active surveillance observations used non‐verbal methods of diarrhoea measurement (e.g. showing carers pictures of stool), and no studies made mention of a ‘gold standard’ of diarrhoea measurement.

Four of the included studies performed direct head‐to‐head comparisons of diarrhoea measurement methods: three examining the effect of differing recall periods on diarrhoea rates, and one examining the effect of questioning frequency on estimated diarrhoea rates. No studies were identified that analysed reactivity in diarrhoea measurement.

### Differences between active and passive surveillance

As stated in the Introduction, the two main categories of disease surveillance are active surveillance (community surveying) and passive surveillance (measures of visits to health facilities). No studies performed direct ‘head‐to‐head’ comparisons of active and passive surveillance. After model‐based adjustment to perform an indirect comparison of passive and active surveillance, passive surveillance was associated with a 97% lower estimated diarrhoea rate than active surveillance (incidence risk ratio [IRR] = 0.03, 95% CI [0.02, 0.06]) (Table 4).

**TABLE 4:** A random effects model showing the association between study characteristics and estimated diarrhoea rates for all observations and only active surveillance observations

		Active vs. Passive		Within active	
		IRR	95% CI	IRR	95% CI
Surveillance type	Reference: Active surveillance Passive surveillance	Ref.	Ref.	Ref.	Ref.
		0.03	(0.02, 0.06)	NA	NA
Region	Reference: Sub‐Saharan Africa	Ref.	Ref.	Ref.	Ref.
	North Africa/Middle East	1.31	(0.44, 3.87)	1.32	(0.45, 3.86)
	Central and South Asia	0.88	(0.13, 6.14)	0.87	(0.13, 6)
	Eastern and South‐East Asia	1.21	(0.47, 3.07)	1.2	(0.47, 3.07)
	Oceania	1.12	(1.05, 1.19)	1.11	(1.05, 1.17)
	South America and Caribbean	0.63	(0.47, 0.84)	0.67	(0.51, 0.9)
Year	Year	0.97	(0.96, 0.98)	0.97	(0.97, 0.98)
Region and year	Reference: Sub‐Saharan Africa	Ref.	Ref.	Ref.	Ref.
	North Africa/Middle East	0.99	(0.95, 1.02)	0.99	(0.95, 1.02)
	Central and South Asia	0.99	(0.93, 1.06)	0.99	(0.93, 1.06)
	Eastern and South‐East Asia	0.99	(0.96, 1.02)	0.99	(0.96, 1.02)
	Oceania	1.00	(1.00, 1.00)	1.00	(1.00, 1.00)
	The Americas	1.02	(1.01, 1.03)	1.02	(1.01, 1.03)
Study design	Reference: Observational primary	Ref.	Ref.	Ref.	Ref.
	Observational secondary	1.05	(0.89, 1.22)	1.04	(0.89, 1.22)
	RCT (baseline or control)	1.19	(0.85, 1.67)	1.5	(1.12, 2.02)
	RCT (experimental post‐intervention)	1.04	(0.75, 1.43)	1.33	(0.98, 1.79)
	Log of measurement period	NA	NA	0.52	(0.46, 0.60)
Questioning frequency	Reference: Daily	NA	NA	NA	NA
	Weekly	NA	NA	1.24	(0.77, 1.99)
	Monthly	NA	NA	2.57	(1.36, 4.84)
	Annually or longer	NA	NA	4.8	(2.73, 8.45)
	One off	NA	NA	4.22	(2.73, 6.52)
Method	Reference: Diary	NA	NA	Ref.	Ref.
	Self‐report	NA	NA	0.85	(0.53, 1.38)
	Constant	7.98	(5.72, 11.14)	13.86	7.3
Random effects parameters	Variance (constant)	1.07	(0.86, 1.33)	0·67	(0.47, 0.95)
	Variance (residual)	0.08	(0.06, 0.11)	0·08	(0.05, 0.11)

### Impacts of factors within active surveillance studies on estimated diarrhoea rates

Specific to active surveillance studies, questioning frequency (how often participants are surveyed), recall period of diarrhoea surveying and retrospective versus prospective questioning may differ.

### Questioning frequency

**FIGURE 2:**
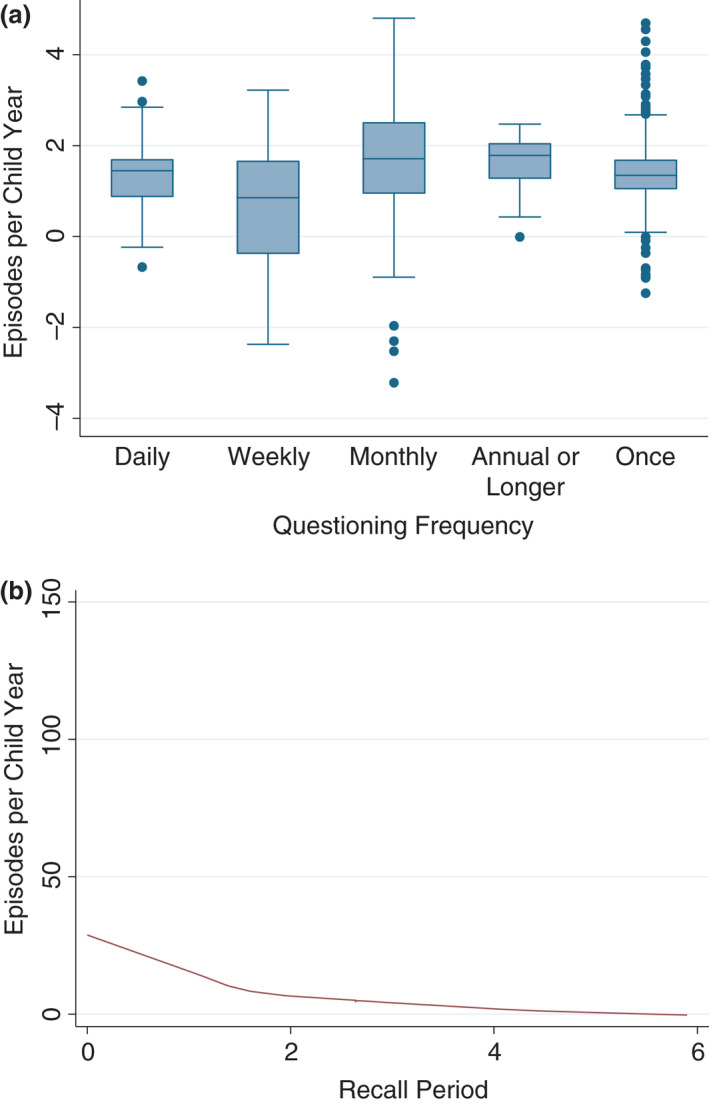
(a) Box plot of estimated diarrhoea rate against questioning frequency for active surveillance studies. (b) Scatter plot and trendline of estimated diarrhoea rates against recall period for active surveillance studies

One study directly examined the effect of differing questioning frequencies on estimated diarrhoea rates; Zwane et al. estimated that biweekly surveys had a 7–15% lower diarrhoea rate than six‐monthly surveys when using the same recall period [21]. Our indirect comparison of active surveillance observations produced comparable results; after model‐based adjustment, we found that less frequent questioning was associated with an increase in estimated diarrhoea rates. For example, one‐time questioning was associated with a rate over four times higher than daily questioning (IRR = 4.22 [2.73, 6.52]) (Table 4). This, however, is not evident graphically in unadjusted crude data – however, a large amount of variance as questioning frequency increases can still be seen (Figure 2a).

### Recall period

Three studies directly examined the effect of differing recall periods on estimated diarrhoea rates, though the recall periods examined were different. Melo et al. found that diarrhoea rates were cut by a third when carers recall over 4 weeks versus 24 h [22]. Feikin et al. similarly estimated that diarrhoea rates were cut by a fifth when carers recall over 11–13 days versus 1–2 days [10]. Lee et al. estimated that estimated diarrhoea rates were similar for carers who recalled over a 72‐h period versus a 24‐h period [23], but this is a much shorter range than that investigated in the other two studies.

Based on an indirect comparison of the included active surveillance observations, we estimated that recall periods and estimated diarrhoea rates were inversely associated. After model‐based adjustment, we found that a doubling of recall period was associated with a 48% reduction in diarrhoea rate (IRR = 0.52 [0.46, 0.60]; Table 4). This is also evident graphically in the crude data (Figure 2b).

### Prospective versus retrospective

No studies directly compared prospective (diary) recall designs against retrospective. We estimated through indirect comparison that retrospective recall observations were associated with a lower rate than prospective (diary) observations, but the effect size was relatively uncertain (IRR = 0.85 [0.53, 1.38]; Table 4).

## DISCUSSION

### Main findings

We provide evidence that estimated under 5 diarrhoea rates are sensitive to the methods used in their measurement. This includes variance introduced by the choice of passive or active surveillance, as well as factors specific to active surveillance.

Passive surveillance methods were associated with 97% lower diarrhoea rates than active surveillance methods. The most probable explanation is that carers do not seek health care for the majority of cases where, if asked, they would report diarrhoea. While not shown in our results, several studies on access to health care among infants in LMICs show that the propensity of carers to seek care for their under 5s with diarrhoea is influenced by diarrhoea severity, socioeconomic or legal status and other demographic characteristics [5, 6, 7, 8, 9].

Regarding active surveillance methods, we found that different questioning frequencies influence estimated diarrhoea rates. There was a trend to lower estimated diarrhoea rates given higher questioning frequencies: one off questioning was associated with an over four times higher estimated diarrhoea rate than daily questioning. We also found that differing recall periods were associated with a change in estimated diarrhoea rates: a doubling of recall period was associated with a halving of estimated diarrhoea rate.

### Factors that result in subjective measurements during active surveillance

As the distinction between and recall of a diarrhoeal or non‐diarrhoeal stool by carers is largely subjective, several cognitive factors can affect measurement. These include respondent fatigue (becoming tired of answering questions), recall bias (forgetting events that have occurred in the past), perception bias (not understanding the question being asked) and reactivity (answering differently due to experiencing an intervention).

### Respondent fatigue

Declining diarrhoea rates with increasing questioning frequency (but the same recall period) suggest respondent fatigue – participants may be inclined to pay attention to their bowel movements at first, but lose motivation with further rounds of questioning.

### Recall bias

Recall bias is the effect of forgetting: participants are more likely to recall recent than older events. We would expect a lower reported number of diarrhoea episodes with longer recall periods and this was borne out by our analysis including two of the three head‐to‐head comparisons – the exception examined a much smaller gap between questions than the other two [10, 22]. This finding was corroborated by a more recent head‐to‐head comparison where daily recall was associated with a 30 percentage point higher estimated diarrhoea rate than fortnightly recall during a text message survey of under 5 diarrhoea in urban Tanzania [24]. While not examined in our review, it has also been reported that the effect of recall bias is more apparent for moderate diarrhoea compared to severe diarrhoea. Zafar et al., for example, found that moderate diarrhoea is reported at half the rate of severe diarrhoea during longer recall periods [11].

### Other factors affecting diarrhoea measurement

Other factors outside the scope of this review can further influence estimated diarrhoea rates. For example, poor caregiver perception of diarrhoea (understanding what is or is not diarrhoea) can result in error in diarrhoea measurement. Voskuijl et al. determined that carers of children under 5 were only able to identify 56–75% of loose or watery stool and 80% healthy stools [12].

Another relevant phenomenon is ‘reactivity’, whereby participants adjust their answers to a survey according to how they believe they ought to respond, regardless of any true underlying difference. We did not identify any studies of reactivity in this review, but it has been discussed as a potential explanatory factor in previous trials. Luby et al. mentioned courtesy bias as a potential source of reactivity, stating ‘people who received the intervention might have been grateful and, out of courtesy, reported less diarrhoea’ [14]. Wood et al. further found evidence for reactivity in clinical trials for various diseases, reporting that inadequate concealment of interventions is associated with improved treatment performance in trials, particularly for subjective outcomes [25]. It was not possible to examine reactivity through courtesy bias or inadequate concealment in our review as WASH trials by nature are unblinded.

## CONCLUSION

The magnitude of the variation in diarrhoea rates, even among active methods of surveillance, suggests the need for standardisation of diarrhoea measurement methods to facilitate comparisons between studies. Despite the 14‐day UNICEF and DHS standard, there does seem to be a trend towards using a 7‐day recall period. It was the most frequently used recall period (51%) among RCTs in our review. Three of the three recent large integrated WASH trials (the SHINE trials in Bangladesh and Kenya, and the WASH Benefits trial in Zimbabwe) also used a 7‐day retrospective recall to measure diarrhoea [26, 27, 28, 29], in contravention of the UNICEF and DHS guidelines. However, the above three trials differed among themselves with respect to question frequency: the SHINE trials questioned carers annually, while the WASH Benefits trial questioned mothers every ‘2 to 6’ months [27, 28, 29].

It could be argued that lack of standardisation simply introduces measurement error in trials that can be counteracted by increasing sample size. However, this is only likely to be true if it is assumed that the different methods affect only the propensity of someone with a true case of diarrhoea (or indeed enteric infection) to report a case of diarrhoea (the ‘sensitivity’ of the method) [30]. If, however, there is a loss of ‘specificity’ – the propensity of someone who did not have diarrhoea (or enteric infection) to report a case of diarrhoea – then intervention effects will also be biased across studies using different methods. It is also likely that any measurement errors will bias results towards the null, rather than towards reports of intervention effectiveness [30]. It is therefore possible that the choice of methodology is at least partially responsible for the widely varying, and often disappointing, results of evaluations on WASH interventions [14, 27, 29, 31, 32].

While a widely accepted standard would facilitate comparisons across different observational and experimental studies, this raises the question of what the optimal standard may be that would also produce reliable reports of diarrhoea rates. There is no ‘gold standard’ method for measurement of diarrhoea rates. In part, this is because of the difficulty in defining the underlying construct and providing a culturally and linguistically consistent definition of a case or episode of ‘diarrhoea’. Direct observation by an expert might constitute a gold standard against which other methods could be compared, as has been described above in the study by Voskuiljl and colleagues [12]. However, judgements among experts may not be universal. Moreover, the collection of every stool and use of experts to classify them quickly becomes impractical at larger scales.

We propose two policies to mitigate the problem. First, an agreed consensus method for the measurement of diarrhoea rates in surveys. Second, triangulation of diarrhoea rates with other observations that reflects on gastrointestinal health when interventions are evaluated. Many WASH studies already include anthropometric measurements as outcomes alongside diarrhoea. Furthermore, direct measurement of environmental contamination and pathogen levels in stool samples should complement diarrhoea rates in clinical studies. This would also allow for determination of how much diarrhoea is attributable to infection (and which can be reduced by WASH interventions), rather than non‐infective reasons (which would likely not be impacted by WASH interventions) [32]. Investigation of the link between interventions, environmental contamination and the profiles of pathogen carriage in childhood stools is an important topic for scientific research.

## APPENDIX 1

### THE NUMBER OF OBSERVATIONS CONTRIBUTED PER STUDY

**Table:**

Number of observations per study	Number of studies (%)
1	175 (60.14)
2	62 (21.31)
3	14 (4.81)
4	19 (6.53)
5	6 (2.06)
6	3 (1.03)
7	2 (0.69)
8	1 (0.34)
10	1 (0.34)
12	1 (0.34)
15	1 (0.34)
16	2 (0.69)
17	1 (0.34)
21	1 (0.34)
34	1 (0.34)

## APPENDIX 2

### SEARCH STRING FOR THE SYSTEMATIC REVIEW

diarrh?ea? or AWD or loose stool or watery stool or dysentery or ABD or fe?cal

Developing Country.**sh**.

(Africa or Asia or Caribbean or West Indies or South America or Latin America or Central America).**hw,ti,ab,cp**.

(Afghanistan or Albania or Algeria or Angola or Antigua or Barbuda or Argentina or Armenia or Armenian or Aruba or Azerbaijan or Bahrain or Bangladesh or Barbados or Benin or Byelarus or Byelorussian or Belarus or Belorussian or Belorussia or Belize or Bhutan or Bolivia or Bosnia or Herzegovina or Hercegovina or Botswana or Brasil or Brazil or Bulgaria or Burkina Faso or Burkina Fasso or Upper Volta or Burundi or Urundi or Cambodia or Khmer Republic or Kampuchea or Cameroon or Cameroons or Cameron or Camerons or Cape Verde or Central African Republic or Chad or Chile or China or Colombia or Comoros or Comoro Islands or Comores or Mayotte or Congo or Zaire or Costa Rica or Cote d'Ivoire or Ivory Coast or Croatia or Cuba or Cyprus or Czechoslovakia or Czech Republic or Slovakia or Slovak Republic or Djibouti or French Somaliland or Dominica or Dominican Republic or East Timor or East Timur or Timor Leste or Ecuador or Egypt or United Arab Republic or El Salvador or Eritrea or Estonia or Ethiopia or Fiji or Gabon or Gabonese Republic or Gambia or Gaza or Georgia Republic or Georgian Republic or Ghana or Gold Coast or Greece or Grenada or Guatemala or Guinea or Guam or Guiana or Guyana or Haiti or Honduras or Hungary or India or Maldives or Indonesia or Iran or Iraq or Isle of Man or Jamaica or Jordan or Kazakhstan or Kazakh or Kenya or Kiribati or Korea or Kosovo or Kyrgyzstan or Kirghizia or Kyrgyz Republic or Kirghiz or Kirgizstan or Lao PDR or Laos or Latvia or Lebanon or Lesotho or Basutoland or Liberia or Libya or Lithuania or Macedonia or Madagascar or Malagasy Republic or Malaysia or Malaya or Malay or Sabah or Sarawak or Malawi or Nyasaland or Mali or Malta or Marshall Islands or Mauritania or Mauritius or Agalega Islands or Mexico or Micronesia or Middle East or Moldova or Moldovia or Moldovian or Mongolia or Montenegro or Morocco or Ifni or Mozambique or Myanmar or Myanma or Burma or Namibia or Nepal or Netherlands Antilles or New Caledonia or Nicaragua or Niger or Nigeria or Northern Mariana Islands or Oman or Muscat or Pakistan or Palau or Palestine or Panama or Paraguay or Peru or Philippines or Philipines or Phillipines or Phillippines or Poland or Portugal or Puerto Rico or Romania or Rumania or Roumania or Russia or Russian or Rwanda or Ruanda or Saint Kitts or St Kitts or Nevis or Saint Lucia or St Lucia or Saint Vincent or St Vincent or Grenadines or Samoa or Samoan Islands or Navigator Island or Navigator Islands or Sao Tome or Saudi Arabia or Senegal or Serbia or Montenegro or Seychelles or Sierra Leone or Slovenia or Sri Lanka or Ceylon or Solomon Islands or Somalia or South Africa or Sudan or Suriname or Surinam or Swaziland or Syria or Tajikistan or Tadzhikistan or Tadjikistan or Tadzhik or Tanzania or Thailand or Togo or Togolese Republic or Tonga or Trinidad or Tobago or Tunisia or Turkey or Turkmenistan or Turkmen or Uganda or Ukraine or Uruguay or USSR or Soviet Union or Union of Soviet Socialist Republics or Uzbekistan or Uzbek or Vanuatu or New Hebrides or Venezuela or Vietnam or Viet Nam or West Bank or Yemen or Yugoslavia or Zambia or Zimbabwe or Rhodesia).**hw,ti,ab,cp**.

((developing or less* developed or under developed or underdeveloped or middle income or low* income or underserved or under served or deprived or poor*) adj (countr* or nation? or population? or world)).**ti,ab**.

((developing or less* developed or under developed or underdeveloped or middle income or low* income) adj (economy or economies)).**ti,ab**.

(low* adj (gdp or gnp or gross domestic or gross national)).**ti,ab**.

(low adj3 middle adj3 countr*).**ti,ab**.

(lmic or lmics or third world or lami countr*).**ti,ab**.

transitional countr*.**ti,ab**.

or/2‐10

measurement or burden or survey or questionnaire or stool sample or collection or (Bristol adj2 scale) or (Amsterdam adj2 scale) or scale or pathogen or microbiological or biological or protein or olfaction or prevalence or incidence or odds or risk

exp animals/ not humans.sh.

1 and 11 and 12

14 not 13

Limit to ((english or french) and yr = "1993 ‐Current")

## APPENDIX 3

### STUDIES INCLUDED IN THE SYSTEMATIC REVIEW

1. a JJ, Davies AR, Smith GD, et al. *Frequency of diarrhoea as a predictor of elevated blood pressure in children*. vol 27. 2009:259–65.
2. a S, Deb AK, Chawla‐Sarkar M, et al. *Factors associated with diarrhoea in young children and incidence of symptomatic rotavirus infection in rural West Bengal*, *India*. 2014;142:1848–58.
3. Abu Mourad TA. Palestinian refugee conditions associated with intestinal parasites and diarrhoea: Nuseirat refugee camp as a case study. *Public Health*. 2004;118(2):131–42.
4. Abu‐Elyazeed RR, Wierzba TF, Frenck RW, et al. Epidemiology of *Shigella*‐associated diarrhea in rural Egyptian children. *American Journal of Tropical Medicine and Hygiene* 2004;71(3):367–72.
5. Abuzerr S, Nasseri S, Yunesian M, et al. Prevalence of diarrheal illness and healthcare‐seeking behavior by age‐group and sex among the population of Gaza strip: a community‐based cross‐sectional study. *BMC Public Health*. 2019;19(1):704.
6. Acharya D, Singh JK, Adhikari M, et al. Association of water handling and child feeding practice with childhood diarrhoea in rural community of Southern Nepal. *Journal of Infection and Public Health*. 2018;11(1):69–74.
7. Adane M, Mengistie B, Kloos H, Medhin G, Mulat W. Sanitation facilities, hygienic conditions, and prevalence of acute diarrhea among under‐five children in slums of Addis Ababa, Ethiopia: Baseline survey of a longitudinal study. *PloS One*. 2017;12(8):e0182783.
8. Agtini MD, Soeharno R, Lesmana M, et al. The burden of diarrhoea, shigellosis, and cholera in North Jakarta, Indonesia: findings from 24 months surveillance. *BMC Infectious Diseases*. 2005;5:89.
9. Agustina R, Sari TP, Satroamidjojo S, Bovee‐Oudenhoven IMJ, Feskens EJM, Kok FJ. Association of food‐hygiene practices and diarrhea prevalence among Indonesian young children from low socioeconomic urban areas. *BMC Public Health*. 2013;13:977.
10. Agustina R, Shankar AV, Ayuningtyas A, Achadi EL, Shankar AH. Maternal agency influences the prevalence of diarrhea and acute respiratory tract infections among young Indonesian children. *Maternal and Child Health Journal*. 2015;19(5):1033–46.
11. Ahiadeke C. Breast‐feeding, diarrhoea and sanitation as components of infant and child health: a study of large scale survey data from Ghana and Nigeria. *Journal of Biosocial Science*. 2000;32(1):47–61.
12. ala N‐B, Emina JB, Nzita PDK, Cappuccio FP. *Diarrhoea*, *acute respiratory infection*, *and fever among children in the Democratic Republic of Congo*. vol 68. 2009:1728–36.
13. ala N‐B, Ji C, Stallard N, Stranges S, Cappuccio FP. *Spatial analysis of risk factors for childhood morbidity in Nigeria*. vol 77. 2007:770–9.
14. ala N‐B, Magadi MA, Madise NJ. *An investigation of district spatial variations of childhood diarrhoea and fever morbidity in Malawi*. vol 62. 2006:1138–52.
15. Alam DS, Marks GC, Baqui AH, Yunus M, Fuchs GJ. Association between clinical type of diarrhoea and growth of children under 5 years in rural Bangladesh. *International Journal of Epidemiology* 2000;29(5):916–21.
16. Alemayehu B, Ayele BT, Valsangiacomo C, Ambelu A. Spatiotemporal and hotspot detection of U5‐children diarrhea in resource‐limited areas of Ethiopia. *Scientific Reports*. 2020;10(1):10997.
17. Ali M, Atkinson D, Underwood P. Determinants of use rate of oral rehydration therapy for management of childhood diarrhoea in rural Bangladesh. *Journal of Health*, *Population*, *and Nutrition*. 2000;18(2):103–8.
18. Aluisio AR, Maroof Z, C, et al. Risk factors associated with recurrent diarrheal illnesses among children in Kabul, Afghanistan: a prospective cohort study. *PloS One*. ;10(2):e0116342.
19. Aluisio AR, Maroof Z, C, et al. Vitamin D3supplementation and childhood diarrhea: a randomized controlled trial. *Pediatrics*. 2013;132(4):e832‐40.
20. Aluko OO, Afolabi OT, Olaoye EA, Adebayo AD, Oyetola SO, Abegunde OO. The management of the faeces passed by under five children: an exploratory, cross‐sectional research in an urban community in Southwest Nigeria. *BMC public health*. 2017;17(1):178.
21. Amugsi DA, Aborigo RA, Oduro AR, Asoala V, Awine T, Amenga‐Etego L. Socio‐demographic and environmental determinants of infectious disease morbidity in children under 5 years in Ghana. *Global health action*. 2015;8:29349.
22. Anders KL, Thompson CN, Thuy NTV, et al. The epidemiology and aetiology of diarrhoeal disease in infancy in southern Vietnam: a birth cohort study. *International journal of infectious diseases*: *IJID*: *official publication of the International Society for Infectious Diseases*. 2015;35:3–10.
23. Anderson LC, Tegegn A, Tessema F, Galea S, r, Hadley C. Food insecurity, childhood illness and maternal emotional distress in Ethiopia. *Public health nutrition*. 2012;15(4):648–55.
24. Anteneh ZA, Andargie K, Tarekegn M. Prevalence and determinants of acute diarrhea among children younger than five years old in Jabithennan District, Northwest Ethiopia, 2014. *BMC public health*. 2017;17(1):99.
25. ari N, Bahl R, Mazumdar S, et al. *Effect of community*‐*based promotion of exclusive breastfeeding on diarrhoeal illness and growth*: *a cluster randomised controlled trial*. Vol 361. 2003:1418–23.
26. ari N, Bahl R, Taneja S, et al. *Substantial reduction in severe diarrheal morbidity by daily zinc supplementation in young north Indian children*. Vol 109. 2002:e86.
27. Arnold BF, Khush RS, Ramaswamy P, et al. A causal framework for evaluating pre‐existing interventions: An example motivated by efforts in the water, sanitation and hygiene sector in rural India. *American Journal of Tropical Medicine and Hygiene*. 2010;83(5):7.
28. Arnold B, Arana B, Mausezahl D, Hubbard A, Colford JM, Jr. Evaluation of a pre‐existing, 3‐year household water treatment and handwashing intervention in rural Guatemala. *International journal of epidemiology*. 2009;38(6):1651–61.
29. Arrizon AV, Andersson N, Ledogar RJ. Micro‐regional planning: evidence‐based community buy‐in for health development in five of Mexico's poorest rural districts. *BMC health services research*. 2011;11:S2.
30. Arvelo W, Montgomery SP, Bryan JP, et al. Incidence and etiology of infectious diarrhea from a facility‐based surveillance system in Guatemala, 2008–2012. *BMC public health*. 2019 2019;19(1):1340.
31. Asling‐Monemi K, Naved RT, Persson LA. Violence against women and increases in the risk of diarrheal disease and respiratory tract infections in infancy: a prospective cohort study in Bangladesh. *Archives of pediatrics & adolescent medicine*. 2009;163(10):931–6.
32. Assis AMO, Barreto ML, Santos LMP, Fiaccone R, da Silva Gomes GS. Growth faltering in childhood related to diarrhea: a longitudinal community based study. *European journal of clinical nutrition*. 2005;59(11):1317–23.
33. Aung T, M, W, Khin HSS, Montagu D. Incidence of pediatric diarrhea and public‐private preferences for treatment in rural Myanmar: a randomized cluster survey. *Journal of tropical pediatrics*. 2013;59(1):10–6.
34. Aung T, Montagu D, Su Su Khin H, et al. Impact of a social franchising program on uptake of oral rehydration solution plus zinc for childhood diarrhea in Myanmar: a community‐level randomized controlled trial. *Journal of tropical pediatrics*. 2014;60(3):189–97.
35. Azage M, Kumie A, Worku A, Bagtzoglou AC. Childhood diarrhea in high and low hotspot districts of Amhara Region, northwest Ethiopia: a multilevel modeling. *Journal of health*, *population*, *and nutrition*. 2016;35:13.
36. Azage M, Kumie A, Worku A, C Bagtzoglou A, Anagnostou E. Effect of climatic variability on childhood diarrhea and its high risk periods in northwestern parts of Ethiopia. *PloS one*. 2017;12(10):e0186933.
37. Bado AR, Susuman AS, Nebie EI. Trends and risk factors for childhood diarrhea in sub‐Saharan countries (1990–2013): assessing the neighborhood inequalities. *Global health action*. 2016 2016;9(1):30166.
38. Banerjee B, Hazra S, B, yopadhyay D. Diarrhea management among under fives. *Indian pediatrics*. 2004;41(3):255–60.
39. Baqui AH, Zaman K, Persson LA, et al. Simultaneous weekly supplementation of iron and zinc is associated with lower morbidity due to diarrhea and acute lower respiratory infection in Bangladeshi infants. *The Journal of nutrition*. 2003;133(12):4150–7.
40. Barakat AA, Nada KH, Ezzat DA. Prevalence and determining factors of anemia and malnutrition among Egyptian children. *Indian journal of medical sciences*. 2013;67(7):168–77.
41. Barreto ML, Milroy CA, Strina A, et al. Community‐based monitoring of diarrhea in urban Brazilian children: incidence and associated pathogens. *Transactions of the Royal Society of Tropical Medicine and Hygiene*. 2006;100(3):234–42.
42. Bawankule R, Singh A, Kumar K, Pedgaonkar S. Disposal of children's stools and its association with childhood diarrhea in India. *BMC public health*. 2017;17(1):12.
43. Becker‐Dreps S, Bucardo F, Vilchez S, et al. Etiology of childhood diarrhea after rotavirus vaccine introduction: a prospective, population‐based study in Nicaragua. *The Pediatric infectious disease journal*. 2014;33(11):1156–63.
44. Becker‐Dreps S, Melendez M, Liu L, et al. Community diarrhea incidence before and after rotavirus vaccine introduction in Nicaragua. *The American journal of tropical medicine and hygiene*. 2013;89(2):246–50.
45. Becker‐Dreps S, Paniagua M, Dominik R, et al. Changes in childhood diarrhea incidence in Nicaragua following 3 years of universal infant rotavirus immunization. *The Pediatric infectious disease journal*. 2011;30(3):243–7.
46. Becquey E, Ouedraogo CT, Hess SY, et al. Comparison of Preventive and Therapeutic Zinc Supplementation in Young Children in Burkina Faso: A Cluster‐Randomized, Community‐Based Trial. *The Journal of nutrition*. 2016;146(10):2058–2066.
47. Bennett A, Epstein LD, Gilman RH, et al. Effects of the 1997–1998 El Nino episode on community rates of diarrhea. *American journal of public health*. 2012;102(7):e63‐9.
48. Binka FN, Anto FK, Oduro AR, et al. Incidence and risk factors of paediatric rotavirus diarrhoea in northern Ghana. *Tropical medicine & international health*: *TM & IH*. 2003;8(9):840–6.
49. Biritwum RB, Asante A, Amoo PK, et al. Community‐based cluster surveys on treatment preferences for diarrhoea, severe diarrhoea, and dysentery in children aged less than five years in two districts of Ghana. *Journal of health*, *population*, *and nutrition*. 2004;22(2):182–90.
50. Bitew BD, Woldu W, Gizaw Z. Childhood diarrheal morbidity and sanitation predictors in a nomadic community. *Italian journal of pediatrics*. 2017;43(1):91.
51. Bizuneh H, Getnet F, Meressa B, Tegene Y, Worku G. Factors associated with diarrheal morbidity among under‐five children in Jigjiga town, Somali Regional State, eastern Ethiopia: a cross‐sectional study. *BMC pediatrics*. 2017;17(1):182.
52. Boadi KO, Kuitunen M. Environmental and health impacts of household solid waste handling and disposal practices in third world cities: the case of the Accra Metropolitan Area, Ghana. *Journal of environmental health*. 2005;68(4):32–6.
53. Boisson S, Schmidt W‐P, Berhanu T, Gezahegn H, Clasen T. Randomized controlled trial in rural Ethiopia to assess a portable water treatment device. *Environmental science & technology*. 2009;43(15):5934–9.
54. Bozkurt AI, Ozgur S, Ozcirpici B. Association between household conditions and diarrheal diseases among children in Turkey: a cohort study. *Pediatrics international*: *official journal of the Japan Pediatric Society*. 2003;45(4):443–51.
55. Brenner JL, Kabakyenga J, Kyomuhangi T, et al. Can volunteer community health workers decrease child morbidity and mortality in southwestern Uganda? An impact evaluation. *PloS one*. 2011;6(12):e27997.
56. Briceno B, Coville A, Gertler P, Martinez S. Are there synergies from combining hygiene and sanitation promotion campaigns: Evidence from a large‐scale cluster‐randomized trial in rural Tanzania. *PloS one*. 2017;12(11):e0186228.
57. Brooks WA, Santosham M, Naheed A, et al. Effect of weekly zinc supplements on incidence of pneumonia and diarrhoea in children younger than 2 years in an urban, low‐income population in Bangladesh: randomised controlled trial. *Lancet (London*, *England)*. 2005;366(9490):999–1004.
58. Brown JM, Proum S, Sobsey MD. *Escherichia coli* in household drinking water and diarrheal disease risk: evidence from Cambodia. *Water science and technology*: *a journal of the International Association on Water Pollution Research*. 2008;58(4):757–63.
59. Cao X, Rawalai K, Thompson AJ, et al. Relationship between feeding practices and weanling diarrhoea in northeast Thailand. *Journal of health*, *population*, *and nutrition*. 2000;18(2):85–92.
60. Capuno JJ, Tan CAR, Jr., Fabella VM. Do piped water and flush toilets prevent child diarrhea in rural Philippines? *Asia*‐*Pacific journal of public health*. 2015;27(2):NP2122‐32.
61. Carlton EJ, Eisenberg JNS, Goldstick J, Cevallos W, Trostle J, Levy K. Heavy rainfall events and diarrhea incidence: the role of social and environmental factors. *American journal of epidemiology*. 2014;179(3):344–52.
62. Cha S, Kang D, Tuffuor B, et al. The Effect of Improved Water Supply on Diarrhea Prevalence of Children under Five in the Volta Region of Ghana: A Cluster‐Randomized Controlled Trial. *International journal of environmental research and public health*. 2015;12(10):12127–43.
63. Cha S, Lee JE, Seo DS, et al. Associations between Household Latrines and the Prevalence of Diarrhea in Idiofa, Democratic Republic of the Congo: A Cross‐Sectional Study. *The American journal of tropical medicine and hygiene*. 2017;97(2):460–468.
64. Checkley W, Epstein LD, Gilman RH, Cabrera L, Black RE. Effects of acute diarrhea on linear growth in Peruvian children. *American journal of epidemiology*. 2003;157(2):166–75.
65. Checkley W, Gilman RH, Black RE, et al. Effect of water and sanitation on childhood health in a poor Peruvian peri‐urban community. *Lancet (London*, *England)*. 2004;363(9403):112–8.
66. Checkley W, Gilman RH, Black RE, et al. Effects of nutritional status on diarrhea in Peruvian children. *The Journal of pediatrics*. 2002;140(2):210–8.
67. Choi SYP. Mechanisms of racial inequalities in prevalence of diarrhoea in South Africa. *Journal of health*, *population*, *and nutrition*. 2003;21(3):264–72.
68. Chowdhury F, Khan IA, Patel S, et al. Diarrheal Illness and Healthcare Seeking Behavior among a Population at High Risk for Diarrhea in Dhaka, Bangladesh. *PloS one*. 2015;10(6):e0130105.
69. Cifuentes E, Mazari‐Hiriart M, Carneiro F, o, Bianchi F, Gonzalez D. The risk of enteric diseases in young children and environmental indicators in sentinel areas of Mexico City. *International journal of environmental health research*. 2002;12(1):53–62.
70. Clasen TF, Brown J, Collin S, Suntura O, Cairncross S, y. Reducing diarrhea through the use of household‐based ceramic water filters: a randomized, controlled trial in rural Bolivia. *The American journal of tropical medicine and hygiene*. 2004;70(6):651–7.
71. Clasen T, Boisson S, Routray P, et al. Effectiveness of a rural sanitation programme on diarrhoea, soil‐transmitted helminth infection, and child malnutrition in Odisha, India: a cluster‐randomised trial. *The Lancet Global health*. 2014;2(11):e645‐53.
72. Coles CL, Seidman JC, Levens J, Mkocha H, Munoz B, West S. Association of mass treatment with azithromycin in trachoma‐endemic communities with short‐term reduced risk of diarrhea in young children. *The American journal of tropical medicine and hygiene*. 2011;85(4):691–6.
73. Colombara DV, H, ez B, et al. Diarrhea Prevalence, Care, and Risk Factors Among Poor Children Under 5 Years of Age in Mesoamerica. *The American journal of tropical medicine and hygiene*. 2016;94(3):544–52.
74. Colombara DV, Hernández B, McNellan CR, et al. Diarrhea prevalence, care, and risk factors among poor children under 5 years of age in Mesoamerica. *The American journal of tropical medicine and hygiene*. 2016 2016;94(3):544–552.
75. Contreras JD, Meza R, Siebe C, et al. Health risks from exposure to untreated wastewater used for irrigation in the Mezquital Valley, Mexico: A 25‐year update. *Water research*. 2017;123:834–850.
76. Crump JA, Mendoza CE, Priest JW, et al. Comparing serologic response against enteric pathogens with reported diarrhea to assess the impact of improved household drinking water quality. *The American journal of tropical medicine and hygiene*. 2007;77(1):136–41.
77. Cupul‐Uicab LA, Terrazas‐Medina EA, H, ez‐Avila M, Longnecker MP. In utero exposure to DDT and incidence of diarrhea among boys from tropical Mexico. *Environmental research*. 2017;159:331–337.
78. Davis WW, Mullany LC, Shwe Oo EK, Richards AK, Iacopino V, Beyrer C. Health and Human Rights in Karen State, Eastern Myanmar. *PloS one*. 2015;10(8):e0133822.
79. Desmennu AT, Oluwasanu MM, John‐Akinola YO, Oladunni O, Adebowale AS. Maternal Education and Diarrhea among Children aged 0–24 Months in Nigeria. *African journal of reproductive health*. 2017;21(3):27–36.
80. Diness BR, Christoffersen D, Pedersen UB, et al. The effect of high‐dose vitamin A supplementation given with bacille Calmette‐Guerin vaccine at birth on infant rotavirus infection and diarrhea: a randomized prospective study from Guinea‐Bissau. *The Journal of infectious diseases*. 2010;202:S243–51.
81. Diouf K, Tabatabai P, Rudolph J, Marx M. Diarrhoea prevalence in children under five years of age in rural Burundi: an assessment of social and behavioural factors at the household level. *Global health action*. 2014;7:24895.
82. do Carmo GMI, Yen C, Cortes J, et al. Decline in diarrhea mortality and admissions after routine childhood rotavirus immunization in Brazil: a time‐series analysis. *PLoS medicine*. 2011;8(4):e1001024.
83. Doocy S, Burnham G. Point‐of‐use water treatment and diarrhoea reduction in the emergency context: an effectiveness trial in Liberia. *Tropical medicine & international health*: *TM & IH*. 2006;11(10):1542–52.
84. Dreibelbis R, Freeman MC, Greene LE, Saboori S, Rheingans R. The impact of school water, sanitation, and hygiene interventions on the health of younger siblings of pupils: a cluster‐randomized trial in Kenya. *American journal of public health*. 2014;104(1):e91‐7.
85. du Preez M, Conroy RM, Ligondo S, et al. Randomized intervention study of solar disinfection of drinking water in the prevention of dysentery in Kenyan children aged under 5 years. *Environmental science & technology*. 2011;45(21):9315–23.
86. e S, Keyzer MA, Arouna A, Sonneveld BGJS. *Addressing diarrhea prevalence in the West African Middle Belt*: *social and geographic dimensions in a case study for Benin*. vol 7. 2008:17.
87. Edwin P, Azage M. Geographical Variations and Factors Associated with Childhood Diarrhea in Tanzania: A National Population Based Survey 2015–16. *Ethiopian journal of health sciences*. 2019 2019;29(4):513–524.
88. El‐Gilany AH, Hammad S. Epidemiology of diarrhoeal diseases among children under age 5 years in Dakahlia, Egypt. *Eastern Mediterranean health journal* 2005;11(4):762–75.
89. Elfadaly HA, Hassanain NA, Hassanain MA, Barakat AM, Shaapan RM. Evaluation of primitive ground water supplies as a risk factor for the development of major waterborne zoonosis in Egyptian children living in rural areas. *Journal of infection and public health*. 2018;11(2):203–208.
90. Elsanousi S, Abdelrahman S, Elshiekh I, et al. A study of the use and impacts of LifeStraw in a settlement camp in southern Gezira, Sudan. *Journal of water and health*. 2009;7(3):478–83.
91. Ercumen A, Arnold BF, Kumpel E, et al. Upgrading a piped water supply from intermittent to continuous delivery and association with waterborne illness: a matched cohort study in urban India. *PLoS medicine*. 2015;12(10):e1001892.
92. Ercumen A, Arnold BF, Naser AM, Unicomb L, Colford JM, Jr., Luby SP. Potential sources of bias in the use of *Escherichia coli* to measure waterborne diarrhoea risk in low‐income settings. *Tropical medicine & international health*: *TM & IH*. 2017;22(1):2–11.
93. Ercumen A, Naser AM, Unicomb L, Arnold BF, Colford JM, Jr., Luby SP. Effects of source‐ versus household contamination of tubewell water on child diarrhea in rural Bangladesh: a randomized controlled trial. *PloS one*. 2015;10(3):e0121907.
94. Escamilla V, Wagner B, Yunus M, Streatfield PK, van Geen A, Emch M. Effect of deep tube well use on childhood diarrhoea in Bangladesh. *Bulletin of the World Health Organization*. 2011;89(7):521–7.
95. Escobar AL, Coimbra CEA, Jr., Welch JR, Horta BL, Santos RV, Cardoso AM. Diarrhea and health inequity among Indigenous children in Brazil: results from the First National Survey of Indigenous People's Health and Nutrition. *BMC public health*. 2015;15:191.
96. Eshete WB. A stepwise regression analysis on under‐five diarrhoael morbidity prevalence in Nekemte town, western Ethiopia: maternal care giving and hygiene behavioral determinants. *East African journal of public health*. 2008;5(3):193–8.
97. Fabiszewski de Aceituno AM, Stauber CE, Walters AR, Meza Sanchez RE, Sobsey MD. A randomized controlled trial of the plastic‐housing BioSand filter and its impact on diarrheal disease in Copan, Honduras. *The American journal of tropical medicine and hygiene*. 2012;86(6):913–21.
98. Fagbamigbe AF, Uthman AO, Ibisomi L. Hierarchical disentanglement of contextual from compositional risk factors of diarrhoea among under‐five children in low‐ and middle‐income countries. *Scientific reports*. 2021 2021;11(1):8564.
99. Fassinou P, Elenga N, Rouet F, et al. Highly active antiretroviral therapies among HIV‐1‐infected children in Abidjan, Cote d'Ivoire. *AIDS (London*, *England)*. 2004;18(14):1905–13.
100. Fawzi WW, Msamanga GI, Wei R, et al. Effect of providing vitamin supplements to human immunodeficiency virus‐infected, lactating mothers on the child's morbidity and CD4+ cell counts. *Clinical infectious diseases*: *an official publication of the Infectious Diseases Society of America*. 2003;36(8):1053–62.
101. Fawzy A, Arpadi S, Kankasa C, et al. Early weaning increases diarrhea morbidity and mortality among uninfected children born to HIV‐infected mothers in Zambia. *The Journal of infectious diseases*. 2011;203(9):1222–30.
102. Feleke H, Medhin G, Abebe A, Beyene B, Kloos H, Asrat D. Enteric pathogens and associated risk factors among under‐five children with and without diarrhea in Wegera District, Northwestern Ethiopia. *The Pan African medical journal*. 2018;29:72.
103. Feleke S, Egata G, Mesfin F, Yilak G, Molla A. Undernutrition and associated factors in orphan children aged 6–59 months in Gambella Southwest, Ethiopia: A community‐based cross‐sectional study. *BMJ Open*. 2021 2021;11(7):e045892.
104. Few R, Lake I, Hunter PR, Tran PG. Seasonality, disease and behavior: using multiple methods to explore socio‐environmental health risks in the Mekong Delta. *Social science & medicine (1982)*. 2013;80:1–9.
105. Francis MR, Sarkar R, Roy S, et al. Effectiveness of membrane filtration to improve drinking water: A quasi‐experimental study from rural southern India. *American Journal of Tropical Medicine and Hygiene*. 2016;95(5):1192–1200.
106. Fuller JA, Clasen T, Heijnen M, Eisenberg JN. Shared sanitation and the prevalence of diarrhea in young children: evidence from 51 countries, 2001–2011. *The American journal of tropical medicine and hygiene*. 2014 2014;91(1):173–180.
107. Gao W, Liu X, Yan H. Prevalence of diarrhea among children less than 36 months of age in rural western China in 2001 and 2005. *The American journal of tropical medicine and hygiene*. 2014;91(6):1197–202.
108. Garrett V, Ogutu P, Mabonga P, et al. Diarrhoea prevention in a high‐risk rural Kenyan population through point‐of‐use chlorination, safe water storage, sanitation, and rainwater harvesting. *Epidemiology and infection*. 2008;136(11):1463–71.
109. Gebru T, Taha M, Kassahun W. Risk factors of diarrhoeal disease in under‐five children among health extension model and non‐model families in Sheko district rural community, Southwest Ethiopia: comparative cross‐sectional study. *BMC public health*. 2014;14:395.
110. Genser B, Strina A, Teles CA, Prado MS, Barreto ML. Risk factors for childhood diarrhea incidence: dynamic analysis of a longitudinal study. *Epidemiology (Cambridge*, *Mass)*. 2006;17(6):658–67.
111. George CM, Perin J, Neiswender de Calani KJ, et al. Risk factors for diarrhea in children under five years of age residing in peri‐urban communities in Cochabamba, Bolivia. *The American journal of tropical medicine and hygiene*. 2014;91(6):1190–6.
112. Geresomo NC, Mbuthia EK, Matofari JW, Mwangwela AM. Targeting caregivers with context specific behavior change training increased uptake of recommended hygiene practices during food preparation and complementary feeding in Dedza district of Central Malawi. *Ecology of food and nutrition*. 2018;57(4):301–313.
113. Ghimire M, Pradhan YV, Maskey MK. Community‐based interventions for diarrhoeal diseases and acute respiratory infections in Nepal. *Bulletin of the World Health Organization*. 2010;88(3):216–21.
114. Gorham TJ, Yoo J, Garabed R, Mouhaman A, Lee J. Water Access, Sanitation, and Hygiene Conditions and Health Outcomes among Two Settlement Types in Rural Far North Cameroon. *International journal of environmental research and public health*. 2017;14(4)
115. Graf J, Meierhofer R, Wegelin M, Mosler H‐J. Water disinfection and hygiene behaviour in an urban slum in Kenya: impact on childhood diarrhoea and influence of beliefs. *International journal of environmental health research*. 2008;18(5):335–55.
116. Graf J, Zebaze Togouet S, Kemka N, Niyitegeka D, Meierhofer R, Gangoue Pieboji J. Health gains from solar water disinfection (SODIS): evaluation of a water quality intervention in Yaounde, Cameroon. *Journal of water and health*. 2010;8(4):779–96.
117. Gundry SW, Wright JA, Conroy RM, et al. Child dysentery in the Limpopo Valley: a cohort study of water, sanitation and hygiene risk factors. *Journal of water and health*. 2009;7(2):259–66.
118. Guo Y‐F, Gan Y‐Y, Guo C‐N, Sun J, Hao L‐P. Nutritional status of under‐five children from urban low‐income families in Xiangtan and Jilin in China. *Journal of Huazhong University of Science and Technology Medical sciences* = *Hua zhong ke ji da xue xue bao Yi xue Ying De wen ban* = *Huazhong keji daxue xuebao Yixue Yingdewen ban*. 2017;37(1):74–78.
119. Gupta DN, Mondal SK, Ghosh S, Rajendran K, Sur D, Manna B. Impact of zinc supplementation on diarrhoeal morbidity in rural children of West Bengal, India. *Acta paediatrica (Oslo*, *Norway*: *1992)*. 2003;92(5):531–6.
120. Habib MA, Soofi S, Sadiq K, et al. A study to evaluate the acceptability, feasibility and impact of packaged interventions ("Diarrhea Pack") for prevention and treatment of childhood diarrhea in rural Pakistan. *BMC public health*. 2013;13:922.
121. Halder AK, Luby SP, Akhter S, Ghosh PK, Johnston RB, Unicomb L. Incidences and Costs of Illness for Diarrhea and Acute Respiratory Infections for Children <5 Years of Age in Rural Bangladesh. *The American journal of tropical medicine and hygiene*. 2017;96(4):953–960.
122. Hansen CL, McCormick BJJ, Azam SI, et al. Substantial and sustained reduction in under‐5 mortality, diarrhea, and pneumonia in Oshikhandass, Pakistan: evidence from two longitudinal cohort studies 15 years apart. *BMC public health*. 2020 2020;20(1):759.
123. Haque R, Mondal D, Kirkpatrick BD, et al. Epidemiologic and clinical characteristics of acute diarrhea with emphasis on *Entamoeba histolytica* infections in preschool children in an urban slum of Dhaka, Bangladesh. *The American journal of tropical medicine and hygiene*. 2003;69(4):398–405.
124. Harding E, Beckworth C, Fesselet J‐F, Lenglet A, Lako R, Valadez JJ. Using lot quality assurance sampling to assess access to water, sanitation and hygiene services in a refugee camp setting in South Sudan: a feasibility study. *BMC public health*. 2017;17(1):643.
125. Harshfield E, Lantagne D, Turbes A, Null C. Evaluating the sustained health impact of household chlorination of drinking water in rural Haiti. *The American journal of tropical medicine and hygiene*. 2012;87(5):786–95.
126. Hartinger SM, Lanata CF, Hattendorf J, et al. Improving household air, drinking water and hygiene in rural Peru: a community‐randomized‐controlled trial of an integrated environmental home‐based intervention package to improve child health. *International journal of epidemiology*. 2016;45(6):2089–2099.
127. Hasan MM, Richardson A. How sustainable household environment and knowledge of healthy practices relate to childhood morbidity in South Asia: analysis of survey data from Bangladesh, Nepal and Pakistan. *BMJ open*. 2017;7(6):e015019.
128. Hassan KE, Mansour A, Shaheen H, et al. The impact of household hygiene on the risk of bacterial diarrhea among Egyptian children in rural areas, 2004–2007. *Journal of infection in developing countries*. 2014;8(12):1541–51.
129. Hatt LE, Waters HR. Determinants of child morbidity in Latin America: a pooled analysis of interactions between parental education and economic status. *Social science & medicine (1982)*. 2006;62(2):375–86.
130. Headey D, Nguyen P, Kim S, Rawat R, Ruel M, Menon P. Is Exposure to Animal Feces Harmful to Child Nutrition and Health Outcomes? A Multicountry Observational Analysis. *The American journal of tropical medicine and hygiene*. 2017;96(4):961–969.
131. Hess SY, Abbeddou S, Jimenez EY, et al. Small‐quantity lipid‐based nutrient supplements, regardless of their zinc content, increase growth and reduce the prevalence of stunting and wasting in young burkinabe children: a cluster‐randomized trial. *PloS one*. 2015;10(3):e0122242.
132. Hipgrave DB, Assefa F, Winoto A, Sukotjo S. Donated breast milk substitutes and incidence of diarrhoea among infants and young children after the May 2006 earthquake in Yogyakarta and Central Java. *Public health nutrition*. 2012;15(2):307–15.
133. Hollm‐Delgado M‐G, Gilman RH, Bern C, et al. Lack of an adverse effect of *Giardia intestinalis* infection on the health of Peruvian children. *American journal of epidemiology*. 2008;168(6):647–55.
134. Hong TK, Dibley MJ, Tuan T. Factors affecting utilization of health care services by mothers of children ill with diarrhea in rural Vietnam. *The Southeast Asian journal of tropical medicine and public health*. 2003;34(1):187–98.
135. Houatthongkham S, Sithivong N, Jennings G, et al. Trends in the incidence of acute watery diarrhoea in the Lao People's Democratic Republic, 2009–2013. *Western Pacific surveillance and response journal*: *WPSAR*. 2016;7(3):6–14.
136. Huda TMN, Unicomb L, Johnston RB, Halder AK, Yushuf Sharker MA, Luby SP. Interim evaluation of a large scale sanitation, hygiene and water improvement programme on childhood diarrhea and respiratory disease in rural Bangladesh. *Social science & medicine (1982)*. 2012;75(4):604–11.
137. Humbwavali JB, Giugliani C, Nunes LN, Dalcastagne SV, Duncan BB. Malnutrition and its associated factors: a cross‐sectional study with children under 2 years in a suburban area in Angola. *BMC public health*. 2019 2019;19(1):220.
138. Hunter PR, Risebro H, Yen M, et al. Water source and diarrhoeal disease risk in children under 5 years old in Cambodia: a prospective diary based study. *BMC public health*. 2013;13:1145.
139. Iannotti LL, Zavaleta N, Leon Z, Caulfield LE. Growth and body composition of Peruvian infants in a periurban setting. *Food and nutrition bulletin*. 2009;30(3):245–53.
140. Imada KS, Araujo TSd, Muniz PT, Padua VLd. Socioeconomic, hygienic, and sanitation factors in reducing diarrhea in the Amazon. *Revista de saude publica*. 2016;50:77.
141. Isenbarger DW, Hien BT, Ha HT, et al. Prospective study of the incidence of diarrhoea and prevalence of bacterial pathogens in a cohort of Vietnamese children along the Red River. *Epidemiology and infection*. 2001;127(2):229–36.
142. Islam M, Ercumen A, Ashraf S, et al. Unsafe disposal of feces of children <3 years among households with latrine access in rural Bangladesh: Association with household characteristics, fly presence and child diarrhea. *PloS one*. 2018;13(4):e0195218.
143. Islam M, Ercumen A, Naser AM, et al. Effectiveness of the Hydrogen Sulfide Test as a Water Quality Indicator for Diarrhea Risk in Rural Bangladesh. *The American journal of tropical medicine and hygiene*. 2017;97(6):1867–1871.
144. Jaeggi T, Kortman GAM, Moretti D, et al. Iron fortification adversely affects the gut microbiome, increases pathogen abundance and induces intestinal inflammation in Kenyan infants. *Gut*. 2015;64(5):731–42.
145. Jensen PK, Ensink JHJ, Jayasinghe G, et al. Effect of chlorination of drinking‐water on water quality and childhood diarrhoea in a village in Pakistan. *Journal of health*, *population*, *and nutrition*. 2003;21(1):26–31.
146. Kaindi DWM, Schelling E, Wangoh JM, Imungi JK, Farah Z, Meile L. Risk factors for symptoms of gastrointestinal illness in rural town Isiolo, Kenya. *Zoonoses and public health*. 2012;59(2):118–25.
147. Kamara JK, Galuk, e M, Maeda F, Luboga S, Renzaho A. Understanding the challenges of improving sanitation and hygiene outcomes in a community based intervention: A cross‐sectional study in rural Tanzania. *International journal of environmental research and public health*. 2017 2017;14(6):602.
148. Kamm KB, Feikin DR, Bigogo GM, et al. Associations between presence of handwashing stations and soap in the home and diarrhoea and respiratory illness, in children less than five years old in rural western Kenya. *Tropical medicine & international health*: *TM & IH*. 2014;19(4):398–406.
149. Kante AM, Gutierrez HR, Larsen AM, et al. Childhood Illness Prevalence and Health Seeking Behavior Patterns in Rural Tanzania. *BMC public health*. 2015;15:951.
150. Kanté AM, Gutierrez HR, Larsen AM, et al. Childhood illness prevalence and health seeking behavior patterns in rural Tanzania. *BMC public health*. 2015 2015;15(1):951.
151. Kariuki JG, Magambo KJ, Njeruh MF, Muchiri EM, Nzioka SM, Kariuki S. Effects of hygiene and sanitation interventions on reducing diarrhoea prevalence among children in resource constrained communities: case study of Turkana District, Kenya. *Journal of community health*. 2012;37(6):1178–84.
152. Kattula D, Francis MR, Kulinkina A, et al. Environmental predictors of diarrhoeal infection for rural and urban communities in south India in children and adults. *Epidemiology and infection*. 2015;143(14):3036–47.
153. Kawakatsu Y, Tanaka J, Ogawa K, Ogendo K, Honda S. Community unit performance: factors associated with childhood diarrhea and appropriate treatment in Nyanza Province, Kenya. *BMC public health*. 2017;17(1):202.
154. Khan MN, Islam MM. Effect of exclusive breastfeeding on selected adverse health and nutritional outcomes: a nationally representative study. *BMC public health*. 2017;17(1):889.
155. Kirby MA, Nagel CL, Rosa G, et al. Use, microbiological effectiveness and health impact of a household water filter intervention in rural Rwanda‐A matched cohort study. *International journal of hygiene and environmental health*. 2017;220(6):1020–1029.
156. Kolahi A‐A, Rastegarpour A, Abadi A, Gachkar L. An unexpectedly high incidence of acute childhood diarrhea in Koot‐Abdollah, Ahwaz, Iran. *International journal of infectious diseases*: *IJID*: *official publication of the International Society for Infectious Diseases*. 2010;14(7):e618‐21.
157. Kolahi A‐A, Rastegarpour A, Sohrabi M‐R. The impact of an urban sewerage system on childhood diarrhoea in Tehran, Iran: a concurrent control field trial. *Transactions of the Royal Society of Tropical Medicine and Hygiene*. 2009;103(5):500–5.
158. Komarulzaman A, Smits J, de Jong E. Clean water, sanitation and diarrhoea in Indonesia: Effects of household and community factors. *Global public health*. 2017;12(9):1141–1155.
159. Kosek M, Yori PP, Pan WK, et al. Epidemiology of highly endemic multiply antibiotic‐resistant shigellosis in children in the Peruvian Amazon. *Pediatrics*. 2008;122(3):e541‐9.
160. Kulinkina A, V. r, Mohan VR, et al. Seasonality of water quality and diarrheal disease counts in urban and rural settings in south India. *Scientific reports*. 2016;6:20521.
161. Kumar S, Vollmer S. Does access to improved sanitation reduce childhood diarrhea in rural India? *Health economics*. 2013;22(4):410–27.
162. Kumi‐Kyereme A, Amo‐Adjei J. Household wealth, residential status and the incidence of diarrhoea among children under‐five years in Ghana. *Journal of epidemiology and global health*. 2016;6(3):131–40.
163. Lee G, Cama V, Gilman RH, et al. Comparison of two types of epidemiological surveys aimed at collecting daily clinical symptoms in community‐based longitudinal studies. *Annals of epidemiology*. 2010;20(2):151–8.
164. Lee G, Yori P, Olortegui MP, et al. Comparative effects of vivax malaria, fever and diarrhoea on child growth. *International journal of epidemiology*. 2012;41(2):531–9.
165. Lee H‐Y, Van Huy N, Choi S. Determinants of early childhood morbidity and proper treatment responses in Vietnam: results from the Multiple Indicator Cluster Surveys, 2000–2011. *Global health action*. 2016;9:29304.
166. Lindquist ED, George CM, Perin J, et al. A cluster randomized controlled trial to reduce childhood diarrhea using hollow fiber water filter and/or hygiene‐sanitation educational interventions. *The American journal of tropical medicine and hygiene*. 2014;91(1):190–7.
167. Long KZ, Montoya Y, Hertzmark E, Santos JI, Rosado JL. A double‐blind, randomized, clinical trial of the effect of vitamin A and zinc supplementation on diarrheal disease and respiratory tract infections in children in Mexico City, Mexico. *The American journal of clinical nutrition*. 2006;83(3):693–700.
168. Long KZ, Rosado JL, DuPont HL, Hertzmark E, Santos JI. Supplementation with vitamin A reduces watery diarrhoea and respiratory infections in Mexican children. *The British journal of nutrition*. 2007;97(2):337–43.
169. Luabeya K‐KA, Mpontshane N, Mackay M, et al. Zinc or multiple micronutrient supplementation to reduce diarrhea and respiratory disease in South African children: a randomized controlled trial. *PloS one*. 2007;2(6):e541.
170. Luby SP, Agboatwalla M, Painter J, Altaf A, Billhimer WL, Hoekstra RM. Effect of intensive handwashing promotion on childhood diarrhea in high‐risk communities in Pakistan: a randomized controlled trial. *JAMA*. 2004;291(21):2547–54.
171. Luby SP, Halder AK, Huda TM, et al. Microbiological Contamination of Drinking Water Associated with Subsequent Child Diarrhea. *The American journal of tropical medicine and hygiene*. 2015;93(5):904–11.
172. Luby SP, Rahman M, Arnold BF, et al. Effects of water quality, sanitation, handwashing, and nutritional interventions on diarrhoea and child growth in rural Bangladesh: a cluster randomised controlled trial. *The Lancet Global health*. 2018;6(3):e302‐e315.
173. Mach O, Lu L, Creek T, et al. Population‐based study of a widespread outbreak of diarrhea associated with increased mortality and malnutrition in Botswana, January‐March, 2006. *The American journal of tropical medicine and hygiene*. 2009;80(5):812–8.
174. Mahmud MA, Hossain MM, Huang DB, Habib M, DuPont HL. Sociodemographic, environmental and clinical risk factors for developing persistent diarrhoea among infants in a rural community of Egypt. *Journal of health*, *population*, *and nutrition*. 2001;19(4):313–9.
175. Malik A, Taneja DK, Devasenapathy N, Rajeshwari K. Short‐course prophylactic zinc supplementation for diarrhea morbidity in infants of 6 to 11 months. *Pediatrics*. 2013;132(1):e46‐52.
176. Manger MS, Taneja S, S, et al. Poor folate status predicts persistent diarrhea in 6‐ to 30‐month‐old north Indian children. *The Journal of nutrition*. 2011;141(12):2226–32.
177. Manna B, Nasrin D, Kanungo S, et al. Determinants of health care seeking for diarrheal illness in young children in urban slums of Kolkata, India. *The American journal of tropical medicine and hygiene*. 2013;89(1):56–61.
178. Mansour A, Shaheen HI, Amine M, et al. Diarrhea burden due to natural infection with enterotoxigenic *Escherichia coli* in a birth cohort in a rural Egyptian community. *Journal of clinical microbiology*. 2014;52(7):2595–603.
179. Marcynuk PB, Flint JA, Sargeant JM, et al. Comparison of the burden of diarrhoeal illness among individuals with and without household cisterns in northeast Brazil. *BMC infectious diseases*. 2013;13:65.
180. Markovitz A, R. a, Goldstick JE, et al. Where science meets policy: comparing longitudinal and cross‐sectional designs to address diarrhoeal disease burden in the developing world. *International journal of epidemiology*. 2012;41(2):504–13.
181. Masangwi SJ, Ferguson NS, Grimason AM, Morse TD, Zawdie G, Kazembe LN. Household and community variations and nested risk factors for diarrhoea prevalence in southern Malawi: a binary logistic multi‐level analysis. *International journal of environmental health research*. 2010;20(2):141–58.
182. Mashal T, Takano T, Nakamura K, et al. Factors associated with the health and nutritional status of children under 5 years of age in Afghanistan: family behaviour related to women and past experience of war‐related hardships. *BMC public health*. 2008;8:301.
183. Mashoto KO, Malebo HM, Msisiri E, Peter E. Prevalence, one week incidence and knowledge on causes of diarrhea: household survey of under‐fives and adults in Mkuranga district, Tanzania. *BMC public health*. 2014;14:985.
184. Mazumder S, Taneja S, Bahl R, et al. Effect of implementation of integrated management of neonatal and childhood illness programme on treatment seeking practices for morbidities in infants: cluster randomised trial. *BMJ (Clinical research ed)*. 2014;349:g4988.
185. Mbonye AK. Prevalence of childhood illnesses and care‐seeking practices in rural Uganda. *TheScientificWorldJournal*. 2003;3:721–30.
186. Mbonye AK. Risk factors for diarrhoea and upper respiratory tract infections among children in a rural area of Uganda. *Journal of health*, *population*, *and nutrition*. 2004;22(1):52–8.
187. Mekasha A, Tesfahun A. Determinants of diarrhoeal diseases: a community based study in urban south western Ethiopia. *East African medical journal*. 2003;80(2):77–82.
188. Melese B, Paulos W, Astawesegn FH, Gelgelu TBA‐G, Temesgen Bati, O. Prevalence of diarrheal diseases and associated factors among under‐five children in Dale District, Sidama zone, Southern Ethiopia: a cross‐sectional study. *BMC public health*. 2019 2019;19(1):1235.
189. Melo MCNd, Taddei JAAC, Diniz‐Santos DR, et al. Incidence of diarrhea in children living in urban slums in Salvador, Brazil. *The Brazilian journal of infectious diseases*: *an official publication of the Brazilian Society of Infectious Diseases*. 2008;12(1):89–93.
190. Melo MCNd, Taddei JAdAC, Diniz‐Santos DR, May DS, Carneiro NB, Silva LR. Incidence of diarrhea: poor parental recall ability. *The Brazilian journal of infectious diseases*: *an official publication of the Brazilian Society of Infectious Diseases*. 2007;11(6):571–9.
191. Mengistie B, Berhane Y, Worku A. Household water chlorination reduces incidence of diarrhea among under‐five children in rural Ethiopia: a cluster randomized controlled trial. *PloS one*. 2013;8(10):e77887.
192. Merga N, Alemayehu T. Knowledge, perception, and management skills of mothers with under‐five children about diarrhoeal disease in indigenous and resettlement communities in Assosa District, Western Ethiopia. *Journal of health*, *population*, *and nutrition*. 2015;33(1):20–30.
193. Mihrshahi S, Ichikawa N, Shuaib M, et al. Prevalence of exclusive breastfeeding in Bangladesh and its association with diarrhoea and acute respiratory infection: results of the multiple indicator cluster survey 2003. *Journal of health*, *population*, *and nutrition*. 2007;25(2):195–204.
194. Moll DM, McElroy RH, Sabogal R, Corrales LF, Gelting RJ. Health impact of water and sanitation infrastructure reconstruction programmes in eight Central American communities affected by Hurricane Mitch. *Journal of water and health*. 2007;5(1):51–65.
195. Molla NA, Ali G, Mollah KA, et al. Migration, health, and socioenvironmental safety net among children of Dhaka, Bangladesh. *Archives of environmental & occupational health*. 2017;72(6):336–342.
196. Mondal D, Haque R, Sack RB, Kirkpatrick BD, Petri WA, Jr. Attribution of malnutrition to cause‐specific diarrheal illness: evidence from a prospective study of preschool children in Mirpur, Dhaka, Bangladesh. *The American journal of tropical medicine and hygiene*. 2009;80(5):824–6.
197. Moore SR, Lima AA, Schorling JB, Barboza MS, Jr., Soares AM, Guerrant RL. Changes over time in the epidemiology of diarrhea and malnutrition among children in an urban Brazilian shantytown, 1989 to 1996. *International journal of infectious diseases*: *IJID*: *official publication of the International Society for Infectious Diseases*. 2000;4(4):179–86.
198. Moraes LR, Cancio JA, Cairncross S, y, Huttly S. Impact of drainage and sewerage on diarrhoea in poor urban areas in Salvador, Brazil. *Transactions of the Royal Society of Tropical Medicine and Hygiene*. 2003;97(2):153–8.
199. Morita T, Perin J, Oldja L, et al. Mouthing of Soil Contaminated Objects is Associated with Environmental Enteropathy in Young Children. *Tropical medicine & international health*: *TM & IH*. 2017;22(6):670–678.
200. Mozumder AB, Barkat EK, Kane TT, Levin A, Ahmed S. The effect of birth interval on malnutrition in Bangladeshi infants and young children. *Journal of biosocial science*. 2000;32(3):289–300.
201. Mshida HA, Kassim N, Kimanya ME, Mpolya E. Influence of Water, Sanitation, and Hygiene Practices on Common Infections among Under‐Five Children in Longido and Monduli Districts of Arusha, Tanzania. *Journal of environmental and public health*. 2017;2017:9235168.
202. Musengimana G, Mukinda FK, Machekano R, Mahomed H. Temperature Variability and Occurrence of Diarrhoea in Children under Five‐Years‐Old in Cape Town Metropolitan Sub‐Districts. *International journal of environmental research and public health*. 2016;13(9)
203. Nakahara S, Poudel KC, Lopchan M, et al. Differential effects of out‐of‐home day care in improving child nutrition and augmenting maternal income among those with and without childcare support: a prospective before‐after comparison study in Pokhara, Nepal. *Health policy (Amsterdam*, *Netherlands)*. 2010;97(1):16–25.
204. Nakamori M, Nguyen XN, Nguyen CK, et al. Nutritional status, feeding practice and incidence of infectious diseases among children aged 6 to 18 months in northern mountainous Vietnam. *The journal of medical investigation*: *JMI*. 2010;57(1):45–53.
205. Ndeezi G, Tylleskar T, Ndugwa CM, Tumwine JK. Multiple micronutrient supplementation does not reduce diarrhoea morbidity in Ugandan HIV‐infected children: a randomised controlled trial. *Paediatrics and international child health*. 2012;32(1):14–21.
206. Negesse Y, Taddese AA, Negesse A, Ayele TA. Trends and determinants of diarrhea among under‐five children in Ethiopia: cross‐sectional study: multivariate decomposition and multilevel analysis based on Bayesian approach evidenced by EDHS 2000–2016 data. *BMC public health*. 2021 2021;21(1):193.
207. Nikiema L, Huybregts L, Martin‐Prevel Y, et al. Effectiveness of facility‐based personalized maternal nutrition counseling in improving child growth and morbidity up to 18 months: A cluster‐randomized controlled trial in rural Burkina Faso. *PloS one*. 2017;12(5):e0177839.
208. Ntshebe O, Channon AA, Hosegood VA‐N, Oleosi, O. Household composition and child health in Botswana. *BMC public health*. 2019 2019;19(1):1621.
209. Oberhelman RA, Gilman RH, Sheen P, et al. An intervention‐control study of corralling of free‐ranging chickens to control Campylobacter infections among children in a Peruvian periurban shantytown. *The American journal of tropical medicine and hygiene*. 2006;74(6):1054–9.
210. Okronipa HET, Marquis GS, Lartey A, Brakohiapa L, Perez‐Escamilla R, Mazur RE. Postnatal depression symptoms are associated with increased diarrhea among infants of HIV‐positive Ghanaian mothers. *AIDS and behavior*. 2012;16(8):2216–25.
211. Olney DK, Pedehombga A, Ruel MT, Dillon A. A 2‐year integrated agriculture and nutrition and health behavior change communication program targeted to women in Burkina Faso reduces anemia, wasting, and diarrhea in children 3–12.9 months of age at baseline: a cluster‐randomized controlled trial. *The Journal of nutrition*. 2015;145(6):1317–24.
212. Omore R, O'Reilly CE, Williamson J, et al. Health care‐seeking behavior during childhood diarrheal illness: results of health care utilization and attitudes surveys of caretakers in western Kenya, 2007–2010. *The American journal of tropical medicine and hygiene*. 2013;89(1):29–40.
213. Opryszko MC, Majeed SW, Hansen PM, et al. Water and hygiene interventions to reduce diarrhoea in rural Afghanistan: a randomized controlled study. *Journal of water and health*. 2010;8(4):687–702.
214. Ortiz J, Van Camp J, Wijaya S, Donoso S, Huybregts L. Determinants of child malnutrition in rural and urban Ecuadorian highlands. *Public health nutrition*. 2014;17(9):2122–30.
215. Osendarp SJ, van Raaij JM, Darmstadt GL, Baqui AH, Hautvast JG, Fuchs GJ. Zinc supplementation during pregnancy and effects on growth and morbidity in low birthweight infants: a randomised placebo controlled trial. *Lancet (London*, *England)*. 2001;357(9262):1080–5.
216. Owusu‐Agyei S, Newton S, Mahama E, et al. Impact of vitamin A with zinc supplementation on malaria morbidity in Ghana. *Nutrition journal*. 2013;12:131.
217. Page A‐L, Hustache S, Luquero FJ, Djibo A, Manzo ML, Grais RF. Health care seeking behavior for diarrhea in children under 5 in rural Niger: results of a cross‐sectional survey. *BMC public health*. 2011;11:389.
218. Pathela P, Zahid Hasan K, Roy E, Huq F, Kasem Siddique A, Bradley Sack R. Diarrheal illness in a cohort of children 0–2 years of age in rural Bangladesh: I. Incidence and risk factors. *Acta paediatrica (Oslo*, *Norway*: *1992)*. 2006;95(4):430–7.
219. Peletz R, S, i M, et al. Drinking water quality, feeding practices, and diarrhea among children under 2 years of HIV‐positive mothers in peri‐urban Zambia. *The American journal of tropical medicine and hygiene*. 2011;85(2):318–26.
220. Pickering AJ, Djebbari H, Lopez C, Coulibaly M, Alzua ML. Effect of a community‐led sanitation intervention on child diarrhoea and child growth in rural Mali: a cluster‐randomised controlled trial. *The Lancet Global health*. 2015;3(11):e701‐11.
221. Piechulek H, Al‐Sabbir A, Mendoza‐Aldana J. Diarrhea and ARI in rural areas of Bangladesh. *The Southeast Asian journal of tropical medicine and public health*. 2003;34(2):337–42.
222. Plate DK, Strassmann BI, Wilson ML. Water sources are associated with childhood diarrhoea prevalence in rural east‐central Mali. *Tropical medicine & international health*: *TM & IH*. 2004;9(3):416–25.
223. Profitos JMH, Mouhaman A, Lee S, et al. Muddying the waters: A new area of concern for drinking water contamination in Cameroon. *International Journal of Environmental Research and Public Health*. 2014;11(12):12454–12472.
224. Prohmmo A, Cook LA, Murdoch DR. Childhood diarrhoea in a district in northeast Thailand: incidence and treatment choices. *Asia*‐*Pacific journal of public health*. 2006;18(2):26–32.
225. Qadri F, Saha A, Ahmed T, Al Tarique A, Begum YA, Svennerholm A‐M. Disease burden due to enterotoxigenic *Escherichia coli* in the first 2 years of life in an urban community in Bangladesh. *Infection and immunity*. 2007;75(8):3961–8.
226. Quadri F, Nasrin D, Khan A, et al. Health care use patterns for diarrhea in children in low‐income periurban communities of Karachi, Pakistan. *The American journal of tropical medicine and hygiene*. 2013;89(1):49–55.
227. Rahman MM, Vermund SH, Wahed MA, Fuchs GJ, Baqui AH, Alvarez JO. Simultaneous zinc and vitamin A supplementation in Bangladeshi children: randomised double blind controlled trial. *BMJ (Clinical research ed)*. 2001;323(7308):314–8.
228. Rao MR, Abu‐Elyazeed R, Savarino SJ, et al. High disease burden of diarrhea due to enterotoxigenic *Escherichia coli* among rural Egyptian infants and young children. *Journal of clinical microbiology*. 2003;41(10):4862–4.
229. Regassa W, Lemma S. Assessment of Diarrheal Disease Prevalence and Associated Risk Factors in Children of 6–59 Months Old at Adama District Rural Kebeles, Eastern Ethiopia, January/2015. *Ethiopian journal of health sciences*. 2016;26(6):581–588.
230. Rego RF, Moraes LRS, Dourado I. Diarrhoea and garbage disposal in Salvador, Brazil. *Transactions of the Royal Society of Tropical Medicine and Hygiene*. 2005;99(1):48–54.
231. Reller ME, Mendoza CE, Lopez MB, et al. A randomized controlled trial of household‐based flocculant‐disinfectant drinking water treatment for diarrhea prevention in rural Guatemala. *The American journal of tropical medicine and hygiene*. 2003;69(4):411–9.
232. Roberts L, Chartier Y, Chartier O, Malenga G, Toole M, Rodka H. Keeping clean water clean in a Malawi refugee camp: a randomized intervention trial. *Bulletin of the World Health Organization*. 2001;79(4):280–7.
233. Rogawski ET, Westreich D, Becker‐Dreps S, et al. The effect of early life antibiotic exposures on diarrheal rates among young children in Vellore, India. *The Pediatric infectious disease journal*. 2015;34(6):583–8.
234. Rollins NC, Ndirangu J, B, et al. Exclusive breastfeeding, diarrhoeal morbidity and all‐cause mortality in infants of HIV‐infected and HIV uninfected mothers: an intervention cohort study in KwaZulu Natal, South Africa. *PloS one*. 2013;8(12):e81307.
235. Ross J, Hanlon C, Medhin G, et al. Perinatal mental distress and infant morbidity in Ethiopia: a cohort study. *Archives of disease in childhood Fetal and neonatal edition*. 2011;96(1):F59‐64.
236. Roushdy R, Sieverding M. Improved water and child health in Egypt: impact of interrupted water supply and storage of household water on the prevalence of diarrhoea. *Eastern Mediterranean health journal* = *La revue de sante de la Mediterranee orientale* = *al*‐*Majallah al*‐*sihhiyah li*‐*sharq al*‐*mutawassit*. 2016;22(1):4–18.
237. Saha D, Akinsola A, Sharples K, et al. Health Care Utilization and Attitudes Survey: understanding diarrheal disease in rural Gambia. *The American journal of tropical medicine and hygiene*. 2013;89(1):13–20.
238. Saha S. Design and baseline findings of a multi‐site non‐randomized evaluation of the effect of a health programme on microfinance clients in India. *Global journal of health science*. 2013;6(1):43–51.
239. Santos CB, Araujo KCGM, Jardim‐Botelho A, et al. Diarrhea incidence and intestinal infections among rotavirus vaccinated infants from a poor area in Brazil: a spatial analysis. *BMC public health*. 2014;14:399.
240. Sarkar R, Gladstone BP, Warier JP, et al. Rotavirus and other Diarrheal Disease in a Birth Cohort from Southern Indian Community. *Indian pediatrics*. 2016;53(7):583–8.
241. Sastry N, Burgard S. The prevalence of diarrheal disease among Brazilian children: trends and differentials from 1986 to 1996. *Social science & medicine (1982)*. 2005;60(5):923–35.
242. Sazawal S, Dhingra U, Dhingra P, et al. Effects of fortified milk on morbidity in young children in north India: community based, randomised, double masked placebo controlled trial. *BMJ (Clinical research ed)*. 2007;334(7585):140.
243. Sazawal S, Dhingra U, Hiremath G, et al. Prebiotic and probiotic fortified milk in prevention of morbidities among children: community‐based, randomized, double‐blind, controlled trial. *PloS one*. 2010;5(8):e12164.
244. Schmidt WP, Boisson S, Routray P, et al. Exposure to cows is not associated with diarrhoea or impaired child growth in rural Odisha, India: a cohort study. *Epidemiology and infection*. 2016;144(1):53–63.
245. Senarath U, Dibley MJ, Agho KE. Breast‐feeding Performance Index: a composite index to describe overall breast‐feeding performance among infants under 6 months of age. *Public health nutrition*. 2007;10(10):996–1004.
246. Shah SM, Yousafzai M, Lakhani NB, Chotani RA, Nowshad G. Prevalence and correlates of diarrhea. *Indian journal of pediatrics*. 2003;70(3):207–11.
247. Sheth M, Obrah M. Diarrhea prevention through food safety education. *Indian journal of pediatrics*. 2004;71(10):879–82.
248. Shivoga WA, Moturi WN. Geophagia as a risk factor for diarrhoea. *Journal of infection in developing countries*. 2009;3(2):94–8.
249. Sima LC, Desai MM, McCarty KM, Elimelech M. Relationship between use of water from community‐scale water treatment refill kiosks and childhood diarrhea in Jakarta. *The American journal of tropical medicine and hygiene*. 2012;87(6):979–84.
250. Simanjuntak CH, Punjabi NH, Wangsasaputra F, et al. Diarrhoea episodes and treatment‐seeking behaviour in a slum area of North Jakarta, Indonesia. *Journal of health*, *population*, *and nutrition*. 2004;22(2):119–29.
251. Soofi S, Cousens S, Iqbal SP, et al. Effect of provision of daily zinc and iron with several micronutrients on growth and morbidity among young children in Pakistan: a cluster‐randomised trial. *Lancet (London*, *England)*. 2013;382(9886):29–40.
252. Sur D, Saha DR, Manna B, Rajendran K, Bhattacharya SK. Periodic deworming with albendazole and its impact on growth status and diarrhoeal incidence among children in an urban slum of India. *Transactions of the Royal Society of Tropical Medicine and Hygiene*. 2005;99(4):261–7.
253. Sur D, Gupta DN, Mondal SK, et al. Impact of zinc supplementation on diarrheal morbidity and growth pattern of low birth weight infants in Kolkata, India: a randomized, double‐blind, placebo‐controlled, community‐based study. *Pediatrics*. 2003;112(6):1327–32.
254. Sur D, Manna B, Deb AK, et al. Factors associated with reported diarrhoea episodes and treatment‐seeking in an urban slum of Kolkata, India. *Journal of health*, *population*, *and nutrition*. 2004;22(2):130–8.
255. Takanashi K, Chonan Y, Quyen DT, Khan NC, Poudel KC, Jimba M. Survey of food‐hygiene practices at home and childhood diarrhoea in Hanoi, Viet Nam. *Journal of health*, *population*, *and nutrition*. 2009;27(5):602–11.
256. Takanashi K, Quyen DT, Le Hoa NT, Khan NC, Yasuoka J, Jimba M. Long‐term impact of community‐based information, education and communication activities on food hygiene and food safety behaviors in Vietnam: a longitudinal study. *PloS one*. 2013;8(8):e70654.
257. Talal AH, Moe CL, Lima AA, et al. Seroprevalence and seroincidence of Norwalk‐like virus infection among Brazilian infants and children. *Journal of medical virology*. 2000;61(1):117–24.
258. Taneja S, B, ari N, et al. Effect of zinc supplementation on morbidity and growth in hospital‐born, low‐birth‐weight infants. *The American journal of clinical nutrition*. 2009;90(2):385–91.
259. Taneja S, S, TA, et al. Folic acid and vitamin B‐12 supplementation and common infections in 6–30‐mo‐old children in India: a randomized placebo‐controlled trial. *The American journal of clinical nutrition*. 2013;98(3):731–7.
260. Tawfeek HI, Najim NH, Al‐Mashikhi S. Efficacy of an infant formula containing anti‐*Escherichia coli* colostral antibodies from hyperimmunized cows in preventing diarrhea in infants and children: a field trial. *International journal of infectious diseases*: *IJID*: *official publication of the International Society for Infectious Diseases*. 2003;7(2):120–8.
261. Thiam S, Diene AN, Fuhrimann S, et al. Prevalence of diarrhoea and risk factors among children under five years old in Mbour, Senegal: a cross‐sectional study. *Infectious diseases of poverty*. 2017;6(1):109.
262. Thiem VD, Schmidt W‐P, Suzuki M, et al. Animal livestock and the risk of hospitalized diarrhoea in children under 5 years in Vietnam. *Tropical medicine & international health*: *TM & IH*. 2012;17(5):613–21.
263. Tornheim JA, M, KB, L, rigan PJ, Cifuentes E. Water privatization, water source, and pediatric diarrhea in Bolivia: epidemiologic analysis of a social experiment. *International journal of occupational and environmental health*. 2009;15(3):241–8.
264. Trudeau J, Aksan A‐M, Vasquez WF. Water system unreliability and diarrhea incidence among children in Guatemala. *International journal of public health*. 2018;63(2):241–250.
265. Tsiko RG. Bayesian spatial analysis of childhood diseases in Zimbabwe. *BMC public health*. 2015;15:842.
266. Turab A, Soofi SB, Ahmed I, Bhatti Z, Zaidi AK, Bhutta ZA. Demographic, socioeconomic, and health characteristics of the MAL‐ED network study site in rural Pakistan. *Clinical Infectious Diseases*. 2014 2014;59:S304–S309.
267. Tylleskar T, Jackson D, Meda N, et al. Exclusive breastfeeding promotion by peer counsellors in sub‐Saharan Africa (PROMISE‐EBF): a cluster‐randomised trial. *Lancet (London*, *England)*. 2011;378(9789):420–7.
268. van der Hoek W, Konradsen F, Ensink JH, Mudasser M, Jensen PK. Irrigation water as a source of drinking water: is safe use possible? *Tropical medicine & international health*: *TM & IH*. 2001;6(1):46–54.
269. van der Hoek W, Feenstra SG, Konradsen F. Availability of irrigation water for domestic use in Pakistan: its impact on prevalence of diarrhoea and nutritional status of children. *Journal of health*, *population*, *and nutrition*. 2002;20(1):77–84.
270. Vasconcelos MJdOB, Rissin A, Figueiroa JN, Lira PICd, Batista Filho M. Factors associated with diarrhea in children under five years old in the state of Pernambuco, according to surveys conducted in 1997 and 2006. *Revista de saude publica*. 2018;52:48.
271. Vasconcelos MJOB, Rissin A, Figueiroa JN, Lira PICd, Batista Filho M. Diarrheal diseases and hospitalization of children under five years of age according to population‐based surveys in the State of Pernambuco, Brazil, in the years 1997 and 2006. *Doencas diarreicas e hospitalizacoes em menores de cinco anos no estado de Pernambuco*, *Brasil*, *nos anos de 1997 e 2006*. 2018;23(3):715–722.
272. Vieira SCF, Gurgel RQ, Kirby A, et al. Acute diarrhoea in a community cohort of children who received an oral rotavirus vaccine in Northeast Brazil. *Memorias do Instituto Oswaldo Cruz*. 2011;106(3):330–4.
273. Volpicelli K, Buttenheim AM. Do Social Factors Predict Appropriate Treatment of Child Diarrheal Disease in Peru? *Maternal and child health journal*. 2016;20(11):2299–2308.
274. Wang X‐Y, Xu Z‐Y, von Seidlein L, et al. Incidence of diarrhea caused by rotavirus infections in rural Zhengding, China: prospective, population‐based surveillance. *The Journal of infectious diseases*. 2005;192:S100–5.
275. Watts H, Gregson S, Saito S, Lopman B, Beasley M, Monasch R. Poorer health and nutritional outcomes in orphans and vulnerable young children not explained by greater exposure to extreme poverty in Zimbabwe. *Tropical medicine & international health*: *TM & IH*. 2007;12(5):584–93.
276. Webb AL, Stein AD, Ramakrishnan U, Hertzberg VS, Urizar M, Martorell R. A simple index to measure hygiene behaviours. *International journal of epidemiology*. 2006;35(6):1469–77.
277. Wierzba TF, El‐Yazeed RA, Savarino SJ, et al. The interrelationship of malnutrition and diarrhea in a periurban area outside Alexandria, Egypt. *Journal of pediatric gastroenterology and nutrition*. 2001;32(2):189–96.
278. Wilson SE, Ouedraogo CT, Prince L, et al. Caregiver recognition of childhood diarrhea, care seeking behaviors and home treatment practices in rural Burkina Faso: a cross‐sectional survey. *PloS one*. 2012;7(3):e33273.
279. Woldemicael G. Diarrhoeal morbidity among young children in Eritrea: environmental and socioeconomic determinants. *Journal of health*, *population*, *and nutrition*. 2001;19(2):83–90.
280. Wu J, Yunus M, Streatfield PK, Emch M. Association of climate variability and childhood diarrhoeal disease in rural Bangladesh, 2000–2006. *Epidemiology and infection*. 2014;142(9):1859–68.
281. Wu J, van Geen A, e, et al. Increase in diarrheal disease associated with arsenic mitigation in Bangladesh. *PloS one*. 2011;6(12):e29593.
282. Wu J, Yunus M, Streatfield PK, et al. Impact of tubewell access and tubewell depth on childhood diarrhea in Matlab, Bangladesh. *Environmental health*: *a global access science source*. 2011;10:109.
283. Xue J, Mhango Z, Hoffman IF, et al. Use of nutritional and water hygiene packages for diarrhoeal prevention among HIV‐exposed infants in Lilongwe, Malawi: an evaluation of a pilot prevention of mother‐to‐child transmission post‐natal care service. *Tropical medicine & international health*: *TM & IH*. 2010;15(10):1156–62.
284. Yassin K. Morbidity and risk factors of diarrheal diseases among under‐five children in rural Upper Egypt. *Journal of tropical pediatrics*. 2000;46(5):282–7.
285. Yotthanooi S, Choonpradub C. A statistical method for estimating under‐reported incidence rates with application to child diarrhea in Thai provinces bordering Cambodia. *The Southeast Asian journal of tropical medicine and public health*. 2010;41(1):203–14.
286. Yu JX, Zhu WP, Ye CC, et al. A cross‐sectional study of acute diarrhea in Pudong, Shanghai, China: prevalence, risk factors, and healthcare‐seeking practices. *Epidemiology and infection*. 2017;145(13):2735–2744.
287. Zavaleta N, Kvistgaard AS, Graverholt G, et al. Efficacy of an MFGM‐enriched complementary food in diarrhea, anemia, and micronutrient status in infants. *Journal of pediatric gastroenterology and nutrition*. 2011;53(5):561–8.
288. Zhang N, Liao W, Yang L, et al. The impact of the 2016 flood event in Anhui Province, China on infectious diarrhea disease: An interrupted time‐series study. *Environment International*. 2019 2019;127:801–809.
